# Identification of novel genes associated with herbicide tolerance in Lentil (*Lens culinaris* ssp. *culinaris* Medik.)

**DOI:** 10.1038/s41598-024-59695-z

**Published:** 2024-05-03

**Authors:** Rind Balech, Fouad Maalouf, Sukhjiwan Kaur, Abdulqader Jighly, Reem Joukhadar, Alsamman M. Alsamman, Aladdin Hamwieh, Lynn Abou Khater, Diego Rubiales, Shiv Kumar

**Affiliations:** 1 International Center for Agricultural Research in the Dry Areas (ICARDA), Terbol, Lebanon; 2 Department of Energy, AgriBio, Environment and Climate Action, Centre for AgriBioscience, 5 Ring Road, Bundoora, VIC 3083 Australia; 3 ICARDA, Cairo, Egypt; 4 Institute for Sustainable Agriculture, CSIC, Córdoba, Spain; 5 ICARDA, New Delhi, India

**Keywords:** Genetic association study, Abiotic

## Abstract

Weeds pose a major constraint in lentil cultivation, leading to decrease farmers’ revenues by reducing the yield and increasing the management costs. The development of herbicide tolerant cultivars is essential to increase lentil yield. Even though herbicide tolerant lines have been identified in lentils, breeding efforts are still limited and lack proper validation. Marker assisted selection (MAS) can increase selection accuracy at early generations. Total 292 lentil accessions were evaluated under different dosages of two herbicides, metribuzin and imazethapyr, during two seasons at Marchouch, Morocco and Terbol, Lebanon. Highly significant differences among accessions were observed for days to flowering (DF) and maturity (DM), plant height (PH), biological yield (BY), seed yield (SY), number of pods per plant (NP), as well as the reduction indices (RI) for PH, BY, SY and NP. A total of 10,271 SNPs markers uniformly distributed along the lentil genome were assayed using Multispecies Pulse SNP chip developed at Agriculture Victoria, Melbourne. Meta-GWAS analysis was used to detect marker-trait associations, which detected 125 SNPs markers associated with different traits and clustered in 85 unique quantitative trait loci. These findings provide valuable insights for initiating MAS programs aiming to enhance herbicide tolerance in lentil crop.

## Introduction

Lentil (*Lens culinaris* ssp. *culinaris* Medik.) is an important legume widely grown in many countries. Being versatile in cooking and a good source of protein as well as various micronutrients, lentil is an essential element for human health and a major component for cereal and rice based alimentary diets^1^. In addition, its straw has a nutritional value as animal feed^2^. Lentil improves soil fertility by its capacity to fix nitrogen and increases soil aeration through its shallow root system^3^.

During the past two decades, the cultivation of lentil has been expanded to new areas by 28% leading to 42% increase in production as well as 18% increase in yield^4^. The yield improvement and cultivation expansion to new regions are the result of the development of appropriate varieties for various market segments and application of good agronomic practices. Despite these achievements, various biotic and abiotic stresses are still affecting its productivity in farmers’ fields such as heat, drought, diseases, and poor weed management. Parasitic and annual broad-leaved weeds cause significant yield losses up to 95%, especially when mismanaged^5,6^. Herbicide tolerance is the most effective technique to control weeds in lentils as other techniques are expensive and time consuming. Sources of tolerance to pre-emergence herbicides (metribuzin and imazethapyr) were identified in lentil^7–9^ and other crops such as faba bean^10^, chickpea^11^, and soybean^12^. Currently, the major efforts for developing herbicide tolerant lentil breeding lines were made through field selection with limited progress due to the low selection accuracy of visual assessment. Improving selection accuracy can be achieved by the utilization of modern breeding methods such as markers assisted selection (MAS) and genomic selection^13^.

Lentil is a diploid (2n = 14) and self-pollinating crop with a large genome size of 4 gigabases (Gb) (Arumuganathan and Earle^14^); its genome is larger than many previously sequenced crops like soybean, chickpea, maize, and rice. However, lentil genome sequencing is possible today due to advances in sequencing technologies and bioinformatics tools^15^. In fact, several linkage maps have been constructed and used for the identification of many genes and quantitative trait loci (QTL) controlling a range of biotic and a biotic traits^16–19^. However, these markers have been proved of limited value due to their narrow association with biparental genetic backgrounds. Genome wide association mapping (GWAS) is an alternative approach that utilize genome-wide single nucleotide polymorphism (SNP) markers for the identification of marker trait associations in diverse germplasm panels^13^. Studies have showed that the optimal implementation of single trait GWAS is under controlled conditions involving one environment and allowing to differentiate between genetic and environmental effect. Moreover, the single trait GWAS doesn’t dissect the presence of correlated traits or pleiotropic effect in contrary to the meta-GWAS approach^20^. However, the results of multiple single-trait GWAS statistics can be combined using meta-GWAS approach^21^ to increase the population size and consequently improve the power of the GWAS analysis^22^. Most meta-GWAS methods required only the SNP effects, calculated using single-trait GWAS for different variants, and their standard errors to calculate a global p-value that is equivalent to the one calculated when combining the actual phenotypic and genotypic data for all variants^23^.

The purpose of this study is to deploy meta-GWAS analysis to identify SNPs markers associated with herbicide damage as well as different agronomic traits of lentil with and without herbicide treatments using multilocation/season phenotypic data.

## Results

### Phenotypic results

#### Herbicide damage score

**Figure 1:**
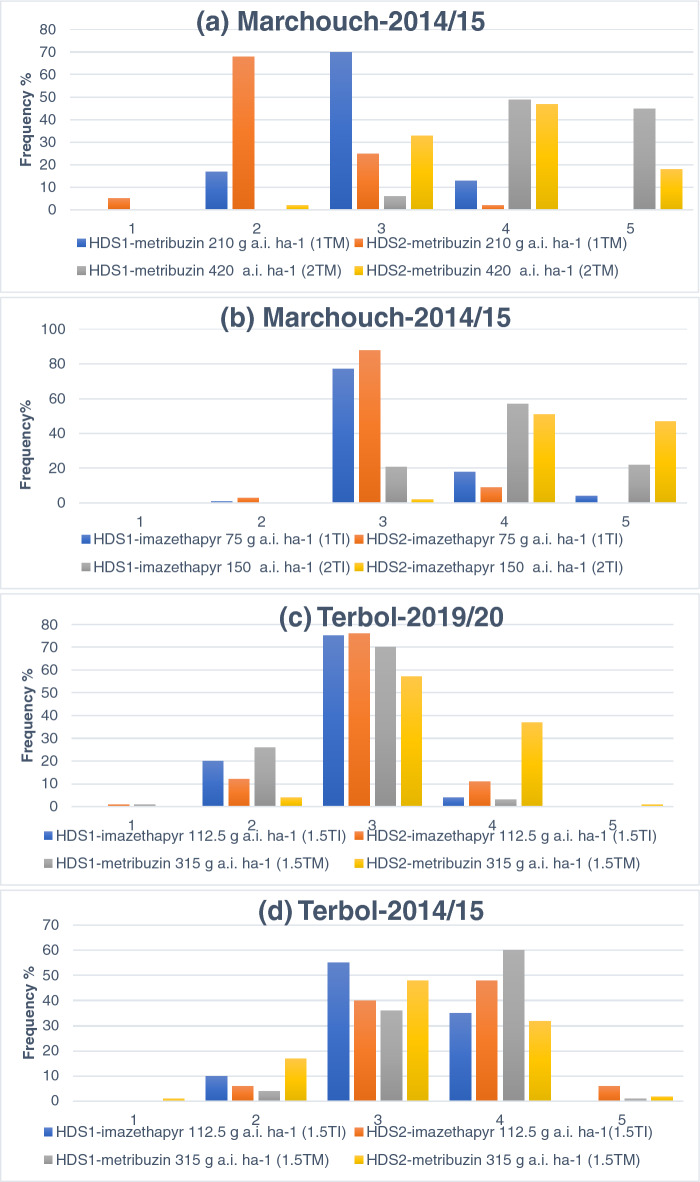
Distribution of lentil genotypes for herbicide damage scores (HDS1 and HDS2) under different dosages of imazethapyr and metribuzin during different locations and cropping seasons.

HDS1 and HDS2 scores ranged from 1 to 5 for imazethapyr and metribuzin at different dosages showing significant variation for herbicide tolerance among the lentil accessions. In Marchouch 2014/15 and after 2 weeks of applying imazethapyr at 75 g a.i.ha^−1^, 1% of the total accessions scored 2 with slight damage on leaves with marginal yellowness, 77% scored 3 with moderate damage with leaf necrosis, 18% scored 4 with severely damaged with 25–75% mortality, and 5% scored 5 with total mortality. HDS2 score taken after 5 weeks of herbicide treatments showed recovery of the injuries in the accessions; 3% of total tested accessions with marginal leaf yellowness recorded 2 score, 88% with moderate damage scored 3, 9% accessions with severe damage scored 4, and no accession scored 5. When applying imazethapyr at 150 g a.i.ha^−1^, HDS1 scored 3 (21% accessions), 4 (57% accessions) and 5 (22% accessions); confirming that the damages were more severe at higher dosage. After 5 weeks of the treatment, HDS2 scored 3, 4 and 5 in 2%, 51% and 47%, of the accessions, respectively showing that no recovery occurred. In Terbol 2014/15 and after 2 weeks of applying imazethapyr at 112.5 g a.i.ha^−1^, HDS1 scored 2 (10% accessions), 3 (55% accessions) and 4 (35% accessions). Whereas HDS2 scored 2 (6% accessions), 3 (40% accessions), 4 (48% accessions) and 5(6% accessions) showing that the toxicity symptoms were aggravated. The observations made during Terbol 2019/20 at the same dosage of imazethapyr (112.5 g a.i.ha^−1^) showed that the toxicity symptoms were less than the symptoms that occurred during Terbol 2014/15 and HDS2 ranged between 1 and 4 showing that the accessions recovered (Fig. 1).

For metribuzin at 210 g a.i.ha^−1^ treatment, HDS1 showed wide variation with 17% of the total accessions scoring 2 with minimum damage (marginal leaf burning), 70% scoring 3 with moderate damage (leaf necrosis and lower vegetative growth), 13% scoring 4 with high damage (severe leaf burning). HDS2 score showed recovery from the herbicide damage with the formation of new leaves. HDS2 score showed that 5% of total accessions scored 1 with no visible damage, 68% scored 2 with slight damage, 25% scored 3 with moderate damage and 2% scored 4 with a mortality rate between 25 and 75%. Similar to imazethapyr, when doubling the dosage of metribuzin (420 g a.i.ha^−1^), HDS1 ranged between 3 and 5 showing aggravation of toxicity symptoms. 5 weeks after the treatment, HDS2 ranged between 2 and 5 showing recovery of the toxicity symptoms. During Terbol 2014/15, when metribuzin (315 g a.i.ha^−1^) was applied, HDS1 scored 2 (10%), 3 (55%) and 4 (35%) while, HDS2 scored 2 (6%), 3 (40%), 4 (48%) and 5 (6%) showing that the toxicity symptoms were aggravated. The observations made during Terbol 2019/20 at the same dosage of metribuzin (315 g a.i.ha^−1^) showed that the toxicity symptoms were less than the ones occurred during Terbol 2014/15 while HDS2 ranged between 2 and 5 showing that the toxicity symptoms aggravated (Fig. 1).

#### Crop phenology, yield and yield components

The combined variance analysis revealed p < 0.001 among the accessions (G) indicating that the tested germplasm was significantly diverse. Moreover, significant differences also existed among treatments (T) and locations (L) for all the traits except for the number of pods per plant (NP). The interaction between genotype × treatment (G × T) across trials and between genotype × Location (G × L) across treatments was also significant. The Genotype × Treatment × Location (G × T × L) interaction showed that the genotypes response to the effect of herbicide treatments was not affected by the environment except for DF and DM and their reduction indexes (Tables 1 and 2). During Terbol 2019/20, plant height (PH) was significantly less in plots treated with imazethapyr at 112.5 g a.i.ha^−1^. Similar observation was observed during Marchouch 2014/15 when treated with imazethapyr at 75 g a.i.ha^−1^ recording a reduction of 28.8% (Tables 3 and 4). The reduction in plant height was severe when imazethapyr was applied at higher dosage (150 g a.i.ha^−1^). When metribuzin applied at 210 g a.i.ha^−1^ and 315 g a.i.ha^−1^ respectively at Marchouch 2014/15 and Terbol 2019/20, the plant height was not significantly reduced in comparison to the untreated plots. On the other hand, when applying Metribuzin at 420 g a.i.ha^−1^, PH was significantly lower (by 33%) than the untreated plots (Tables 3 and 4).

**Table 1:** Combined analysis performed for detecting differences among genotypes (G), Environment (E), Treatments (T) and G × T, G × E and G × T × E interactions for phenological and agronomic traits performed for the trials at Marchouch during 2014/15 and at Terbol during 2014/15 and 2019/20.

	DF	DM	PH	BY	SY	NP
Genotype (G)	< 0.001	< 0.001	< 0.001	< 0.001	< 0.001	< 0.001
Treatment (T)	< 0.001	< 0.001	< 0.001	< 0.001	< 0.001	0.07
G × T	< 0.001	< 0.001	0.003	0.023	0.04	0.06
Environment (E)	< 0.001	< 0.001	< 0.001	< 0.001	< 0.001	ND
G × E	< 0.001	< 0.001	< 0.001	< 0.001	< 0.001	ND
G × T × E	< 0.001	< 0.001	0.936	0.987	1	ND

*DF* days to 50% flowering, *DM* days to maturity, *PH* plant height, *BY* biological yield per plant, *SY* seed yield per plant, *NP* number of pods per plant, *ND* not defined.

**Table 2:** Combined analysis performed for detecting differences among genotype (G), Environment (E), Treatment (T) and G × T, G × E and G × T × E interactions for reduction indexes (RI) of phenological and agronomic traits performed for the trials at Marchouch during 2014/15 and at Terbol during 2014/15 and 2019/20.

	RI_DF_	RI_DM_	RI_PH_	RI_BY_	RI_SY_	RI_NP_
Genotype (G)	0.9	0.6	< 0.001	< 0.001	< 0.001	< 0.001
Treatment (T)	< 0.001	< 0.001	< 0.001	< 0.001	0.02	0.4
Environment (E)	ND	ND	< 0.001	< 0.001	< 0.001	ND
G × E	ND	ND	< 0.001	0.1	0.2	ND

*RI*_*DF*_ reduction index for days to 50% flowering, *RI*_*DM*_ RI for days to maturity, *RI*_*PH*_ RI for plant height, *RI*_*BY*_ RI for biological yield per plant, *RI*_*SY*_ RI for seed yield per plant, *RI*_*NP*_ RI for number of pods per plant, *ND* not defined.

**Table 3:** Mean ± standard error (SE) for different traits under different environments and treatments.

Treatment	DF	DM	PH	BY	SY
Terbol-2014/15					
Imazethapyr at 112.5 g a.i.ha^−1^	124.8 ± 8.7	168.2 ± 5.5	17.7 ± 4.6	1.7 ± 0.8	0.2 ± 0.2
Metribuzin at 315 g a.i.ha^−1^	119.5 ± 8.7	166.6 ± 5.5	18.6 ± 4.6	1.5 ± 0.8	0.3 ± 0.2
Terbol-2019/20					
Imazethapyr at 112.5 g a.i.ha^−1^	162 ± 9.1	207.4 ± 2.8	27 ± 3.9	3.4 ± 1.5	0.8 ± 0.6
Metribuzin at 315 g a.i.ha^−1^	133.1 ± 9.1	194.2 ± 2.8	29 ± 3.9	2.5 ± 1.5	0.9 ± 0.6
No herbicide treatment	131.7 ± 9.1	191.2 ± 2.8	35.5 ± 3.9	3.5 ± 1.5	1.3 ± 0.6
Marchouch-2014/15					
Imazethapyr at 75 g a.i.ha^−1^	ND	ND	26.3 ± 5.5	3.6 ± 2	0.3 ± 0.5
Imazethapyr at 150 g a.i.ha^−1^	ND	ND	20.2 ± 5.5	1.8 ± 2	0.02 ± 0.5
Metribuzin at 210 g a.i.ha^−1^	ND	ND	31.2 ± 5.5	4.4 ± 2	0.3 ± 0.5
Metribuzin at 420 g a.i.ha^−1^	ND	ND	24.6 ± 5.5	1.9 ± 2	0.1 ± 0.5
No herbicide treatment	ND	ND	37.6 ± 5.5	3.7 ± 2	0.2 ± 0.5

*SE* standard error, *ND* Not defined, *DF* days to 50% flowering, *DM* days to maturity, *PH* plant height, *BY* biomass per plant, *SY* seed weight per plant.

**Table 4:** Mean ± Standard error (SE) for reduction indexes (RI) of different traits under different environments and treatments.

Treatment	RI_PH_	RI_BY_	RI_SY_	RI_DF_	RI_DM_	RI_NP_	RI_NS_
Terbol-2019/20							
Imazethapyr at 112.5 g a.i.ha^−1^	22.8 ± 13.77	− 12 ± 65.9	25.1 ± 61.8	− 9.6 ± 15.3	− 3.2 ± 21.5	− 30.6 ± 308.5	20 ± 70.6
Metribuzin at 315 g a.i.ha^−1^	16.6 ± 13.77	15.6 ± 65.9	15.4 ± 61.8	− 0.3 ± 15.3	5.6 ± 21.5	− 6.4 ± 308.5	14.2 ± 70.6
Marchouch-2014/15							
Imazethapyr at 75 g a.i.ha^−1^	28.8 ± 18.2	6.6 ± 94.1	16.3 ± 98.6	ND	ND	ND	ND
Imazethapyr at 150 g a.i.ha^−1^	45.1 ± 18.2	78.2 ± 94.1	101.7 ± 98.6	ND	ND	ND	ND
Metribuzin at 210 g a.i.ha^−1^	15.9 ± 18.2	− 25.7 ± 94.1	2.4 ± 98.6	ND	ND	ND	ND
Metribuzin at 420 g a.i.ha^−1^	33 ± 18.2	58 ± 94.1	63.3 ± 98.6	ND	ND	ND	ND

*SE* standard error, *ND* not defined, *RI*_*DF*_ days to 50% flowering, *RI*_*DM*_ days to maturity, *RI*_*PH*_ plant height, *RI*_*BY*_ biological yield per plant, RI_SY_ seed yield per plant, *RI*_*NP*_ number of pods per plant, *RI*_*NS*_ number of seeds per plant.

During Terbol 2019/20, the biological yield per plant (BY) when treated with imazethapyr at 112.5 g a.i.ha^−1^ or metribuzin at 315 g a.i.ha^−1^ was not significantly lower than the untreated plots. Same observation was obtained during Marchouch 2014/15 when treated with different dosages of imazethapyr and metribuzin. However, when applying imazethapyr at 150g a.i.ha^−1^ or metribuzin at 420 g a.i.ha^−1^, the reduction of BY (RI_BY_) increased to 78.2% and 58% Respectively. Similar results were obtained for seed yield per plant (SY) when treated with imazethapyr or metribuzin at both locations Terbol and Marchouch. When increasing the dosage of imazethapyr at 150 g a.i.ha^−1^ or metribuzin at 420 g a.i.ha^−1^, the reduction in SY (RI_SY_) increased to 101.7% and 63.3% respectively.

### Genotyping and population structure

**Figure 2:**
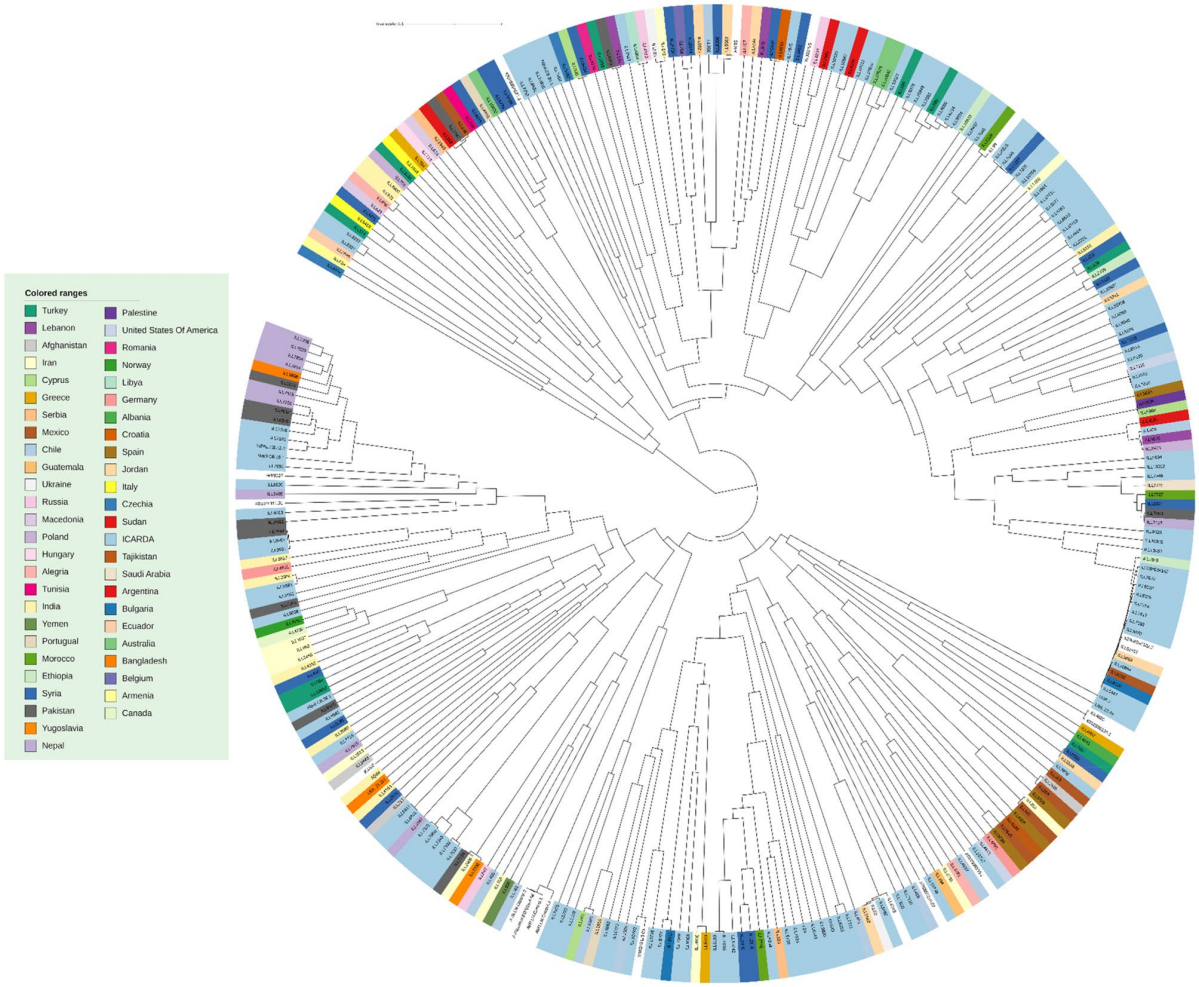
Phylogenetic tree of the studied lentil accessions using SNP genotyping data. The samples are color-coded based on their country of origin.

After applying the quality control criteria, the final dataset consisted of 7642 SNPs that were distributed along the lentil genome. The proportions of sequence variations of the SNP markers are as follows: A/C (1433 SNPs), A/G (3675 SNPs), C/T (3764 SNPs), and G/T (1399 SNPs). The aim of our study was to use phylogenetic diversity to investigate the genetic relationship among this set of lentil population. Through the obtained phylogenetic tree, we identified three significant clusters that evenly accommodated the lentil accessions under investigation, but we did not observe any clustering of genotypes based on their country of origin (Fig. 2).

### GWAS and annotation analyses

**Figure 3:**
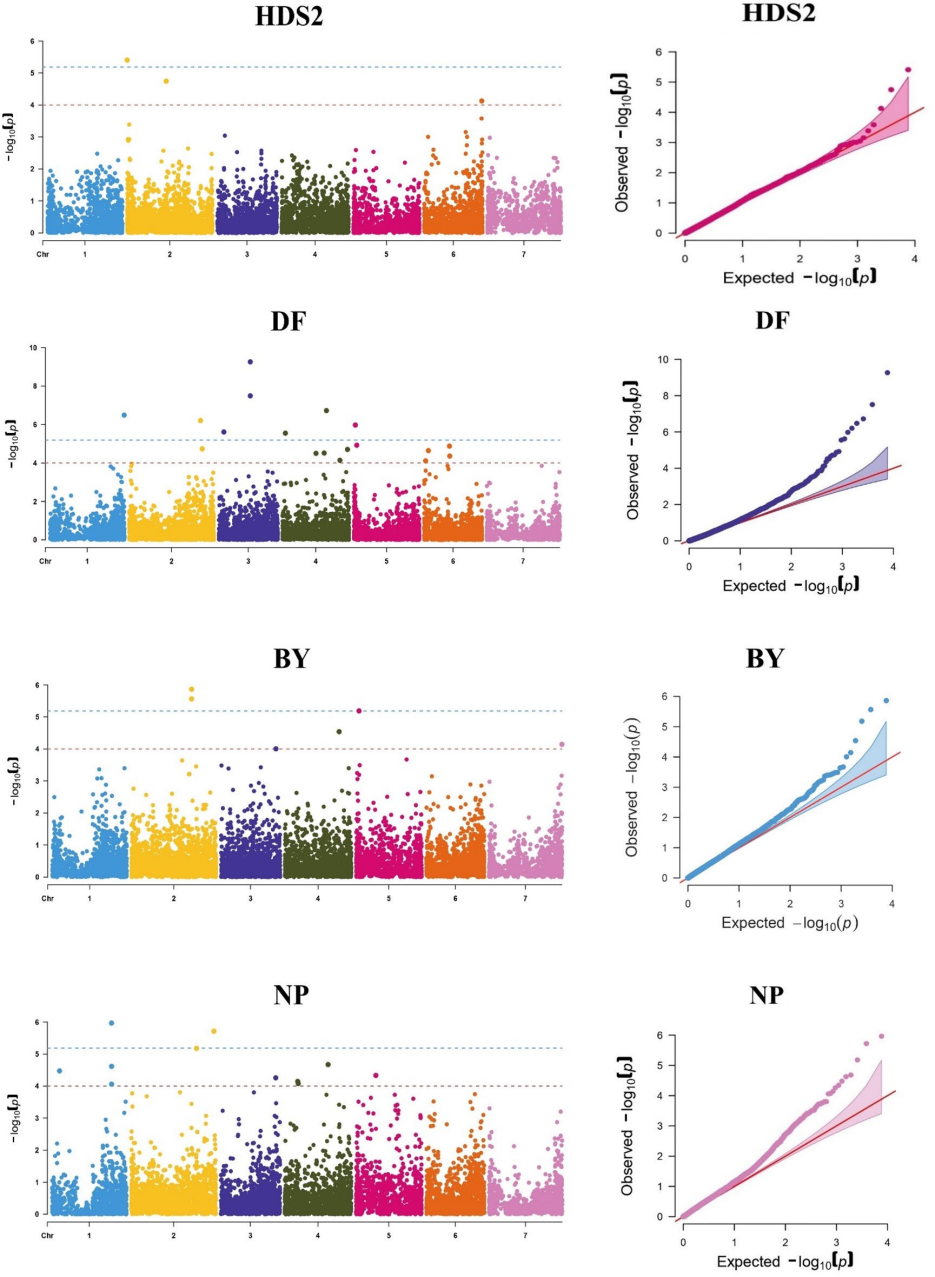

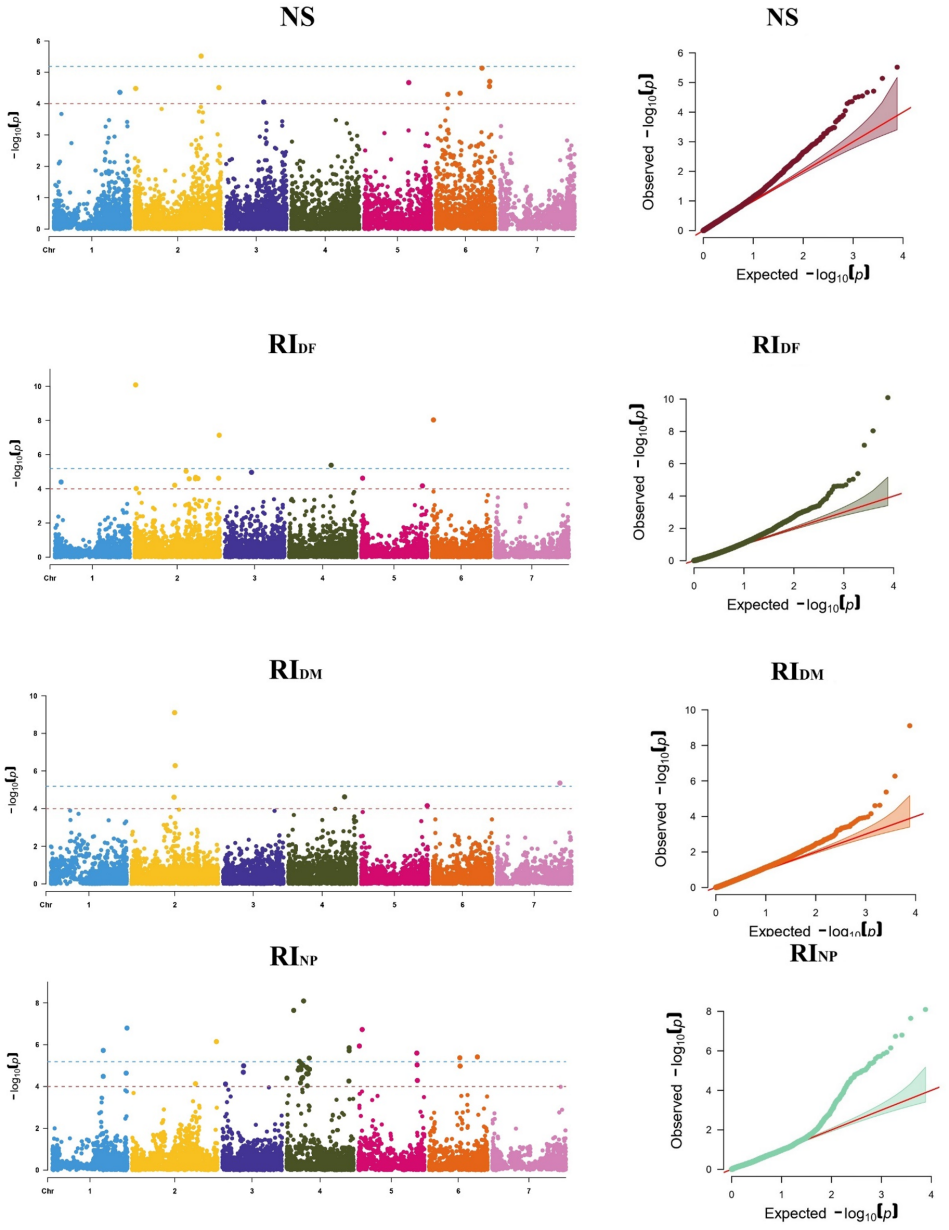
Manhattan plot and QQ plot of the highly significant associations existing between the SNP markers of the recorded traits. HDS2: second herbicide damage score, DF: days to flowering, RIDF: DF reduction index, RIDM: Days to maturity reduction index, BY: Biological yield per plant, NP: number of pods per plant, RINP: NP reduction index, NS: number of seeds per plant.

Among the 7642 SNP markers that were assessed, 125 (clustered in 85 unique QTL) were found to be associated with herbicide tolerance and other traits, of which 36 (clustered in 30 unique QTL) were highly significant (Table 5) while the remaining SNPs were considered as suggestive associations (see Supplementary Table S3 online). Remarkably, traits like RIPH, RIBY, and RISY were excluded as there was no SNP associated with herbicide tolerance. Based on Bonferroni threshold (0.05/n) correction at p > 4.6 × 10^−6^, The SNPs with − log 10 (p value) ≥ 5.2 were considered to have significant associations; 36 SNPs markers were significantly highly associated with diverse traits. Table 5 describes the positions and the significance of these SNP markers for the recorded traits as following: one SNP (AVR-Lc-01885.02–000,213,238) was associated with HDS (− log10(p) = 6.3), eight SNPs (the most significant are AVR-Lc-03987.03–231,578,053, AVR-Lc-03983.03–230,295,656 and AVR-Lc-05801.04–318,079,189) were associated with DF (− log10(p) = 5.6 to 9.3), four SNPs (AVR-Lc-03458.02–007,762,915, AVR-Lc-08010.06–011,899,372, AVR-Lc-03379.02–609,257,610 and AVR-Lc-05740.04–302,184,757) were associated with RI_DF_ (− log10(p) = 5.4 to 8), three SNPs (AVR-Lc-02189.02–307,011,079, AVR-Lc-02200.02–309,350,505 and AVR-Lc-10007.07–447,269,681) with RI_DM_ (− log10(p) = 5.4 to 9.1), three SNPs (AVR-Lc-02714.02–436,766,259, AVR-Lc-02715.02–436,994,699, and AVR-Lc-06969.05–022,095,933) with BY (− log10(p) = 5.2 to 5.9), two SNPs (AVR-Lc-00835.01–430,931,278 and AVR-Lc-03296.02–599,856,144) were associated with NP (− log10(p) = 5.7 and 6.0), and fourteen SNPs (the most significant SNPs are AVR-Lc-01352.01–535,793,448, AVR-Lc-06527.04–050,552,783, and AVR-Lc-05203.04–122,734,802) were associated with RI_NP_ (− log10(p) = 5.2 to 8.1). The significance of associations and the location on the chromosomes of these SNP are also presented in Manhattan plot and QQ plot (Fig. 3). Manhattan plot showed that SNP markers were dispersed randomly on the chromosomes from 1 to 7.

**Table 5:** Highly significant SNP-Trait associations revealed by the Meta-GWAS analysis.

QTL	SNP	Chr	− log10(p)	MAF
HDS2				
QTL013	AVR-Lc-01885.02–000,213,238	2	6.3	0.409
DF				
QTL012	AVR-Lc-01367.01–537,371,482	1	6.5	0.077
QTL026	AVR-Lc-02988.02–518,425,731	2	6.2	0.100
QTL039	AVR-Lc-03983.03–230,295,656	3	7.5	0.074
QTL039	AVR-Lc-03987.03–231,578,053	3	9.3	0.074
QTL034	AVR-Lc-04656.03–038,315,503	3	5.6	0.120
QTL044	AVR-Lc-05454.04–021,398,009	4	5.6	0.056
QTL051	AVR-Lc-05801.04–318,079,189	4	6.7	0.072
QTL061	AVR-Lc-06725.05–011,593,595	5	6.0	0.090
RI_DF_				
QTL032	AVR-Lc-03379.02–609,257,610	2	7.1	0.085
QTL014	AVR-Lc-03458.02–007,762,915	2	10.1	0.050
QTL049	AVR-Lc-05740.04–302,184,757	4	5.4	0.073
QTL071	AVR-Lc-08010.06–011,899,372	6	8.0	0.053
RI_DM_				
QTL019	AVR-Lc-02189.02–307,011,079	2	9.1	0.104
QTL019	AVR-Lc-02200.02–309,350,505	2	6.3	0.130
QTL083	AVR-Lc-10007.07–447,269,681	7	5.4	0.368
BY				
QTL021	AVR-Lc-02714.02–436,766,259	2	5.6	0.071
QTL021	AVR-Lc-02715.02–436,994,699	2	5.9	0.084
QTL062	AVR-Lc-06969.05–022,095,933	5	5.2	0.071
NP				
QTL007	AVR-Lc-00835.01–430,931,278	1	6.0	0.062
QTL029	AVR-Lc-03296.02–599,856,144	2	5.7	0.089
RI_NP_				
QTL006	AVR-Lc-00579.01–366,322,027	1	5.7	0.136
QTL011	AVR-Lc-01352.01–535,793,448	1	6.8	0.142
QTL031	AVR-Lc-03373.02–608,709,301	2	6.2	0.092
QTL047	AVR-Lc-05203.04–122,734,802	4	8.1	0.063
QTL047	AVR-Lc-05322.04–163,251,061	4	5.4	0.080
QTL057	AVR-Lc-06339.04–451,674,618	4	5.8	0.071
QTL057	AVR-Lc-06341.04–451,714,023	4	5.7	0.062
QTL045	AVR-Lc-06527.04–050,552,783	4	7.6	0.094
QTL047	AVR-Lc-06652.04–092,451,825	4	5.2	0.080
QTL064	AVR-Lc-07086.05–026,835,995	5	6.7	0.060
QTL067	AVR-Lc-07474.05–420,462,935	5	5.6	0.071
QTL060	AVR-Lc-07900.05–008,235,645	5	5.9	0.089
QTL076	AVR-Lc-08292.06–221,642,340	6	5.4	0.070
QTL077	AVR-Lc-08717.06–348,699,147	6	5.4	0.077
NS				
QTL025	AVR-Lc-02857.02–473,502,035	2	5.5	0.133

*QTL* quantitative trait loci, *SNP* single nucleotide polymorphism, *Chr* Chromosome, *MAF* minor allele frequency, *HDS2* second herbicide damage score, *DF* days to flowering, *RI*_*D*F_ DF reduction index, RI_DM_ days to maturity reduction index, *BY* biological yield per plant, *NP* number of pods per plant, *RI*_*NP*_ NP reduction index, *NS* number of seeds per plant.

### Physical map and gene annotation

**Figure 4:**
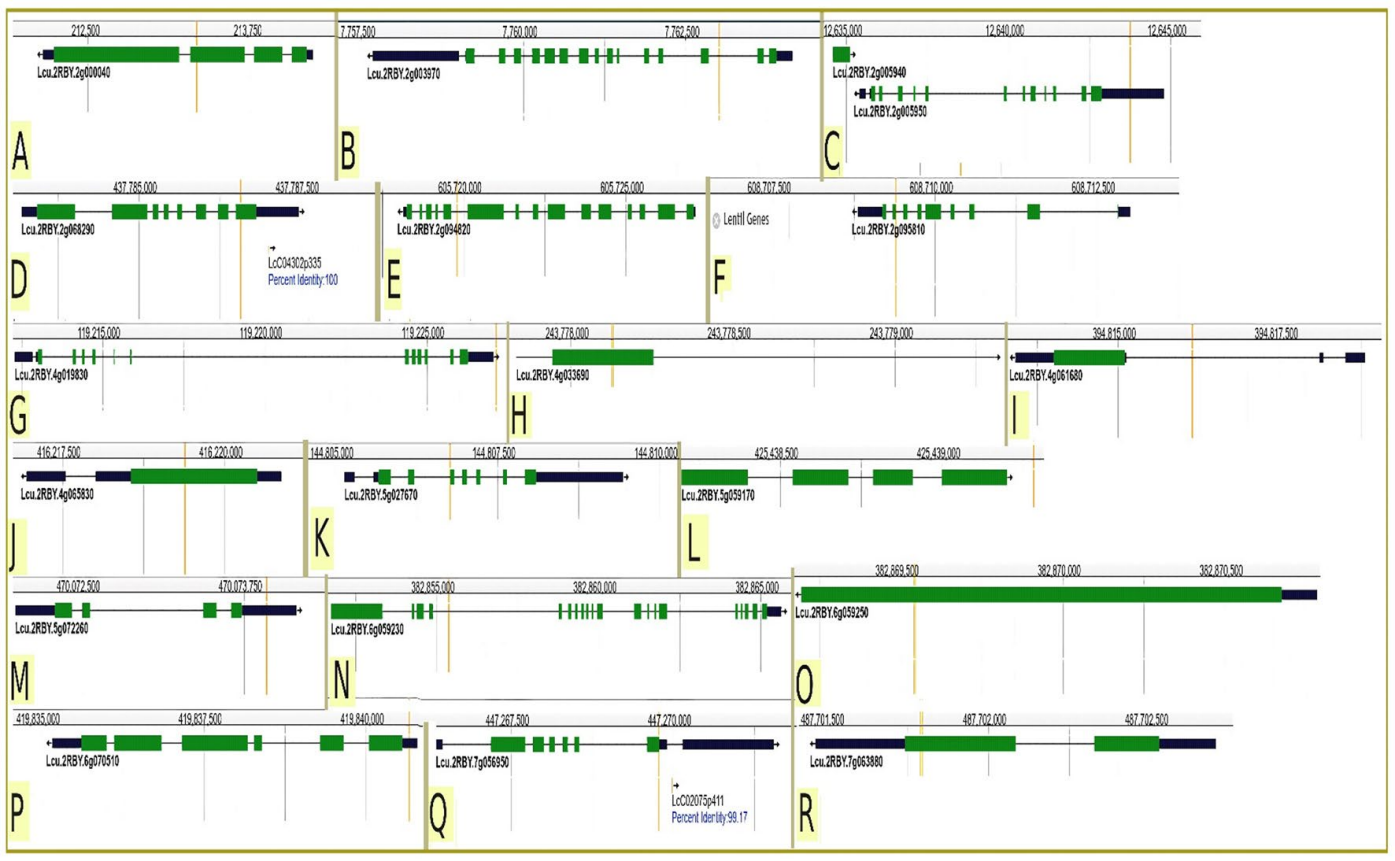
Physical map showing eighteen SNP markers (A to R) that are located on the genes. Green zones are the exomes (coded regions) interrupted by the black zones that are the introns (non-coding regions) and the yellow vertical line represents the location of SNP markers of interest.

The physical map presents the SNPs that are located on the genes which are composed of exons (coded regions) and interrupted by introns (non-coding regions) (Fig. 4). Out of the eighteen SNPs (A to R) that were found located on the genes, only nine SNPs (A, D, F, H, J, K, O, Q and R) were located on the exomes whereas the rest were found located on the introns on chromosomes 2, 4, 5, 6, and 7.

Based on the table of gene annotation, out of the eighteen SNPs that are located inside the gene, four SNPs (AVR-Lc-01885.02–000,213,238, AVR-Lc-03458.02–007,762,915, AVR-Lc-03373.02–608,709,301, and AVR-Lc-10007.07–447,269,681) were found highly associated with herbicide tolerance (Table 6). Gene annotation showed that SNP AVR-Lc-01885.02–000,213,238 highly associated with HDS2 (− log10(p) = 6.3) is located on chromosome 2 within a gene annotated Peptide and nitrate transporter type I and II extracellular region ABC transporter related, SNP AVR-Lc-03458.02–007,762,915 is highly associated with RI_DF_ (− log10(p) = 10.1) is located on chromosome 2 within a gene annotated Allantoinase and Dihydroorotase, SNP AVR-Lc-03373.02–608,709,301 highly associated with RI_NP_ (− log10(p) = 6.2) and located on chromosome 2 within a gene annotated Biotin carboxyl carrier acetyl-CoA carboxylase, and SNP AVR-Lc-10007.07–447,269,681 highly associated with RI_DM_ (− log10(p) = 5.4) is located on chromosome 7 within a gene annotated Myelodysplasia-myeloid leukemia factor 1-interacting protein. Nevertheless, only SNPs AVR-Lc-01885.02–000,213,238, AVR-Lc-03373.02–608,709,301, and AVR-Lc-10007.07–447,269,681 were found located on the exomes (coded regions) (Fig. 4).

**Table 6:** Gene annotation table showing the herbicide tolerance SNP marker and the associated gene and their location; in red are the SNPs detected highly associated with herbicide tolerance.

SNP code	SNP name	Marker location	Gene location	Gene name	Description
**A**	AVR-Lc-01885.02–000213238	Lcu.2RBY.Chr2:213138..213338	Lcu.2RBY.Chr2:2126..214236	Lcu.2RBY.2g000040	Peptide and nitrate transporter type I and II extracellular region ABC transporter related
**B**	AVR-Lc-03458.02–007762915	Lcu.2RBY.Chr2:7762815..7763015	Lcu.2RBY.Chr2:7757679..7764148	Lcu.2RBY.2g003970	Allantoinase and Dihydroorotase
C	AVR-Lc-01558.02–012643639	Lcu.2RBY.Chr2:12643539..12643739	Lcu.2RBY.Chr2:12635412..12644807	Lcu.2RBY.2g005950	Maternal effect embryo arrest 18 protein
D	AVR-Lc-02719.02–437786468	Lcu.2RBY.Chr2:437786368..437786568	Lcu.2RBY.Chr2:437783190..437787471	Lcu.2RBY.2g068290	Receptor-like kinase
E	AVR-Lc-03341.02–605719692	Lcu.2RBY.Chr2:605719592..605719792	Lcu.2RBY.Chr2:605718142..605727161	Lcu.2RBY.2g094820	Aleurone layer morphogenesis protein
**F**	AVR-Lc-03373.02–608709301	Lcu.2RBY.Chr2:608709201..608709401	Lcu.2RBY.Chr2:608708810..608713013	Lcu.2RBY.2g095810	Biotin carboxyl carrier acetyl-CoA carboxylase
G	AVR-Lc-05192.04–119227034	Lcu.2RBY.Chr4:119226934..119227134	Lcu.2RBY.Chr4:119212289..119227063	Lcu.2RBY.4g019830	Nitric oxide synthase-associated protein
H	AVR-Lc-05529.04–243778031	Lcu.2RBY.Chr4:243777931..243778131	Lcu.2RBY.Chr4:243777832..243779307	Lcu.2RBY.4g033690	Putative uncharacterized protein
I	AVR-Lc-06116.04–399656942	Lcu.2RBY.Chr4:399656842..399657042	Lcu.2RBY.Chr4:394813417..394818809	Lcu.2RBY.4g061680	DUF1644 family protein
J	AVR-Lc-06170.04–416219290	Lcu.2RBY.Chr4:416219190..416219390	Lcu.2RBY.Chr4:416216947..416220874	Lcu.2RBY.4g065830	F-box plant protein putative
K	AVR-Lc-06798.05–144806665	Lcu.2RBY.Chr5:144806565..144806765	Lcu.2RBY.Chr5:144805139..144809439	Lcu.2RBY.5g027670	AMSH-like ubiquitin thioesterase
L	AVR-Lc-07494.05–425439183	Lcu.2RBY.Chr5:425439083..425439283	Lcu.2RBY.Chr5:425438196..425439199	Lcu.2RBY.5g059170	Eukaryotic translation initiation factor 2c
M	AVR-Lc-07771.05–470073827	Lcu.2RBY.Chr5:470073727..470073927	Lcu.2RBY.Chr5:470071986..470074154	Lcu.2RBY.5g072260	Sterol carrier protein putative
N	AVR-Lc-08869.06–382855264	Lcu.2RBY.Chr6:382855164..382855364	Lcu.2RBY.Chr6:382851739..382865635	Lcu.2RBY.6g059230	PHD zinc finger protein
O	AVR-Lc-08870.06–382869448	Lcu.2RBY.Chr6:382869348..382869548	Lcu.2RBY.Chr6:382869193..382870784	Lcu.2RBY.6g059250	Glycosyltransferase
P	AVR-Lc-09103.06–419840563	Lcu.2RBY.Chr6:419840463..419840663	Lcu.2RBY.Chr6:419835158..419840798	Lcu.2RBY.6g070510	L-fucokinase and GDP-L-fucose pyrophosphorylase
**Q**	AVR-Lc-10007.07–447269681	Lcu.2RBY.Chr7:447269581..447269781	Lcu.2RBY.Chr7:447266340..447271550	Lcu.2RBY.7g056950	Myelodysplasia-myeloid leukemia factor 1-interacting protein
R	AVR-Lc-10129.07–487701700	Lcu.2RBY.Chr7:487701600..487701800	Lcu.2RBY.Chr7:487701468..487702702	Lcu.2RBY.7g063880	Early nodulin-like protein

## Discussion

In Mediterranean environments of cool winters, lentil has slow growth and crop development, which motivates weeds to compete for water, nutrients, sunlight, and space and hosts diseases and pests that causes severe yield losses in this crop^5,8^. It has been reported that imazethapyr and metribuzin are effective to control weeds when applied to herbicide tolerant lentil accessions. Sources of tolerance to both herbicides were detected in lentils by Balech et al.^7,24^ and Sharma et al.^8,9^ which allowed them to escape phytotoxicity symptoms caused by the herbicides.

In this study, phytotoxicity symptoms were observed when applying imazethapyr or metribuzin herbicides. The herbicide damage score evaluated the degree of phenotypic phytotoxicity, and considerable variability was observed in the phenotypic response. The recovery or aggravation of the phytotoxicity symptoms is subject to the potential of accessions to metabolize the herbicides and detoxify the plants^25^. Additionally, the phenology of the tested accessions was also impacted by delaying the flowering and maturity dates of some lentil accessions and caused a reduction in yield and its components. Similar results were obtained in lentil^7–9, 24^, faba bean^10^, and chickpea^26,27^. Consequently, the phytotoxicity symptoms were ascribed to the inhibition of photosynthesis and plant growth caused by these herbicides as obtained Sharma et al.^8^.

The phylogenetic tree analysis didn’t discern any specific pattern of genotypes based on their country of origin. Therefore, we suggest the possibility of seed exchange occurrence between countries. Thus, it appears that lentils possess broad genetic diversity that is not particular to a specific geographic location because of long-term seed migration and trading across borders.

Limited progress has been made in identifying lentil cultivars tolerant to herbicides through conventional breeding methods, especially that these approaches have been proven to be relatively slow in achieving considerable advances. Hence, it is mandatory to develop genetic markers linked to traits associated with herbicide tolerance in lentils in order to enhance selection accuracy and facilitate early-stage selection. These markers serve as effective tools for selecting adapted and tolerant accessions. Many studies have proved that GWAS is the most successful tool in identifying significant SNPs and candidate genes related to various traits. However, there is a limited number of GWAS reports conducted on lentil such as aphanomyces root rot resistance^28^, prebiotic carbohydrates^29^, anthracnose resistance^30^, ascochyta blight resistance^31^ and seed protein and amino acids content^32^. Compared to other crops like maize and sorghum, the development of genetic resources for lentil has been relatively slower. Nevertheless, new horizons in next generation sequencing (NGS) technologies will open as the lentil genome has been recently published^15,33^.

The MetaGWAS method that was applied in the present study, was initially employed in human genetics as it is impossible to gather multi-environmental data for the same population^34,35^. Its effectiveness over standard GWAS analysis was proved, which encouraged its usage in crops^35,36^. In fact, standard GWAS is more powerful when experiments are conducted under controlled conditions^37–39^. Moreover, conducting experiments in the same environment for a diverse set of accessions that are intended to be grown all over the world can lead to an improper image of the environmental effects on the genetics of the tested set. Many quantitative traits are raw measurements collected from different environments; if standard GWAS analysis is applied, bias effect may be caused which will negatively affect the detection of significant QTL^40^. Therefore, Meta-analysis is an adequate alternative to bypass the previously mentioned challenges of standard GWAS. In our case, MetaGWAS was the best option to be applied since we have an unbalanced set of data collected on 292 accessions, with different treatments on two different locations and two different cropping seasons with a total sample size of 11,956. This approach was also applied by Shook et al.^41^ on a sample of 17,556 accessions of soybean from 73 published studies, by Joukhadar et al.^35^ on a sample of 2571 accessions of wheat, by Battenfield et al.^42^ on wheat with a total sample size of 4095 and Fikere et al.^43^ on a sample of 585 canola accessions. To the best of our knowledge, this is the first MetaGWAS study applied in lentil crops and targeting QTL associated with herbicide tolerance. Hence, most of the identified QTL in this study appear to be new and have not been reported previously.

Based on the physical map results, four SNPs were detected located on the gene and found highly associated with the recorded traits relative to herbicide tolerance. The associations and mechanisms of tolerance to herbicides between the detected SNPs markers on the genomic regions and the phenotypic traits have been deciphered in the following.

The Peptide and nitrate transporter type I and II extracellular region ABC transporter related protein, belongs to the ATP binding cassette (ABC) transporters family and was detected and found associated with herbicide damage score (HDS). This protein transports amino acids, peptides, and nitrate through the plant’s cell membrane using the energy of ATP hydrolysis^44–46^. Several studies have proved that plants have the highest diversity of ABC transporters genes such as in Arabidopsis and in rice with 120 and 121 coding sequences respectively^47,48^. Some of the ABC transporters are responsible for the defence mechanisms to biotic and abiotic stresses and others are involved in the basic functions indispensable for plant growth^49^. Furthermore, Van Eerd et al.^50^ acknowledged that this enzyme is typically associated with herbicide metabolism and plant detoxification. Moreover, genes encoding for ABC transporters proteins were also detected in wheat (*Triticum aestivum* L.)^51,52^, *Arabidopsis thaliana*^53^, and soybean^54^, and performed the function of detoxification of plants from imazethapyr and metribuzin. In this study, the HDS discerned the recovery of some accessions from phytotoxicity symptoms after imazethapyr or metribuzin treatments which might be due to the role of detoxification executed by ABC transporters.

Allantoinase and Dihydroorotase proteins belong to the same superfamily of amidohydrolases^55^; they were detected and found highly associated with the RI_DF_. They participate in various stages of plant development through the de novo pathway by using simple molecules such as CO_2_, amino acids and tetrahydrofolate to build purine and pyrimidine nucleotides^56,57^. Imazethapyr and metribuzin have indirect effect on Allantoinase and Dihydroorotase proteins; Imazethapyr disrupts amino acids synthesis and metribuzin (triazine herbicide) inhibits tetrahydrofolate synthesis^58^. In addition, Duran^59^ and Kafer^60^ reported that flowering stage required the presence of high concentrations of Purine and Pyrimidine. In rice^61^ and in *Arabidopsis thaliana*^60^, the genes encoding to purine and pyrimidine metabolism were responsible for the tolerance to the stress that might encounter the plants during the flowering stage. In this study, when either of both herbicides was applied, the flowering stage was delayed for some accessions but not for others. This observation might be explained by the differing concentrations of purine and pyrimidine available in the plants especially during the flowering stage which depends on the lentil variety and its level of tolerance to the applied herbicide.

Biotin carboxyl carrier (BCC) and acetyl-CoA carboxylase proteins (ACC) were detected and found highly associated with RI_NP_. BCC is used by the enzyme biotin carboxylase to form carboxybiotin that is transferred to ACC enzyme (ACCase). ACCase engender the carboxylation of acetyl-CoA to form malonyl-CoA; essential for fatty acid synthesis and other secondary compounds such as flavonoids^62^. This enzyme plays an essential role in embryo morphogenesis and in apical meristem development^63^. This explains the detected association with the reduction index of number of pods (RI_NP_) in this study much likely as has been reported in *Arabidopsis thaliana*^62^ and *Populus simonii*^64^. Moreover, ACCase plays a role in biotic and abiotic stress tolerance in plants. Many studies like in lentil^65^, *Brassica napus*^66,67^ *Arabidopsis thaliana*^68^, and tobacco^69^ showed that plants can improve their resilience to stress by stimulating the accumulation of ACCase and consequently improving the seed yield. This explains the different levels of tolerance to the applied herbicides expressed in the RI_NP_.

Myelodysplasia-myeloid leukemia factor 1-interacting protein was found to be highly associated with RI_DM_ in this study. It is encoded by (MLF1IP) gene is a transcription factor that was first detected in mammals and *Drosophila*^70^. MLF1IP interacts as a transcriptional repressor with MLF1 and nucleophosmin-MLF1 (NPM-MLF1) to prevent apoptosis (programmed cell death), and thus facilitating cell growth and proliferation in different cell types^71^. As far as we can tell, very rare are the studies that report the presence of MLF1IP in plants and this is the first study that reports its presence in lentils. This gene was also found in tea *Camellia sinensis*, but limited information is available online (A database of gene co-expression network for tea plant (*Camellia sinensis*)). Thus, the function of MLF1IP in plants remains to be elucidated, but since they are transcription factors then their role is to regulate cell death triggered by abiotic and biotic stresses^72,73^.

Moreover, several studies have reported that herbicides cause oxidative stress in plants similar to other abiotic stresses^74,75^. This idea highlights the hypothesis that herbicide tolerance in lentils could result from several mechanisms enabling plants to tolerate the stress caused by herbicide treatment very similar to their response to other abiotic stresses. Thus, the tolerance observed in this study is attributed to the mechanisms that significantly contribute to the detoxification of herbicides in lentil crops.

## Conclusion

Weed management in lentil has become crucial for attempting high yields and good quality to meet the growing global demand. Therefore, the natural genetic variability that lentil crop accessions have shown in previous studies encouraged us to screen a large germplasm collection to search for more powerful and diverse sources for post-emergence herbicide tolerance. This will promote the use of herbicide tolerant varieties with conservation agriculture systems at a lower cost on the farmers. But this method of traditional screening for herbicide tolerance in the field is time consuming, very costly, and hectic. Therefore, genomic selection and marker-assisted selection for herbicide tolerance will greatly improve precision and efficiency of breeding for herbicide tolerance and will help plant breeders in accelerating the breeding process. In this study, we identified four SNP markers that were highly associated with traits related to imazethapyr and metribuzin tolerance using the meta-GWAS method. These identified SNPs could be studied further and used to facilitate selection in breeding programs.

## Materials and methods

### Materials and experiments

A set of 292 lentil accessions including 175 landraces collected from 49 countries, and 117 breeding lines developed at ICARDA were evaluated to their response to imazethapyr and metribuzin treatments, separately at different doses (Supplementary Table S1).

Four field experiments were conducted at Marchouch, Morocco (33.56°N, 6.69°W) during 2013/14 and 2014/15 and at Terbol, Lebanon (33.81°N, 35.98°E) during 2014/15^7^ and 2019/20 (Supplementary Fig. S2), in alpha lattice design with two replicates and a plot size of 1 row of 1 m length spaced at 0.3 m distance. Different dosages of imazethapyr and metribuzin and control treatment (no-herbicide treatment) were applied separately at both locations Marchouch and Terbol at the pre-flowering stage (5–6th node stage). The details of each experiment and the applied treatments are presented in Table 7.

**Table 7:** Environmental conditions of different location-season-treatment combinations of lentil screening.

Location-season	Treatment	Soil type	Rainfall (mm)	Air temperature (°C)		
				average (AVG)	AVG min	AVG max
Marchouch-2013/14	Imazethapyr 37.5 (g a.i.ha^−1^)	Vertisols and silty clay	248	16.5	8.7	20.1
	Imazethapyr 75 (g a.i.ha^−1^)					
	Imazethapyr 112.5 (g a.i.ha^−1^)					
Marchouch-2014/15	Imazethapyr 75 (g a.i.ha^−1^)	Vertisols and silty clay	291	14.1	8.5	19.7
	Imazethapyr 150 (g a.i.ha^−1^)					
	Metribuzin 210 (g a.i.ha^−1^)					
	Metribuzin 420 (g a.i.ha^−1^)					
	No herbicide treatment					
Terbol-2014/15	Imazethapyr 112.5 (g a.i.ha^−1^)	Clay loam	421	9.8	2.8	16.9
	Metribuzin 315 (g a.i.ha^−1^)					
Terbol-2019/20	Imazethapyr 112.5 (g a.i.ha^−1^)	Clay loam	671	10.2	3.5	17.1
	Metribuzin 315 (g a.i.ha^−1^)					
	No herbicide treatment					

### Phenotypic data for herbicide tolerance

Based on the Lentil ontology^76^ the following phenotypic data were recorded:

*Herbicide* *damage* *score* *(HDS)* was recorded using the scale described in Balech et al.^7^ on a scale of 1–5, at 2 weeks (HDS1) and then at 5 weeks (HDS2) after the herbicide application at Terbol in 2014/15 but at Marchouch in 2013/14, only HDS2 was recorded. This scale was proposed by Gaur et al.^11^ to assess the ability of accessions to recover from herbicide treatments.

*Crop* *phenology* *traits* of number of days to 50% flowering (DF) and days to 95% of maturity (DM) from sowing day were recorded on a plot basis at Terbol in 2014–15 and 2019/20.

*Agronomical* *and* *yield* *traits* of plant height (PH) (cm), biological yield/plant (BY) (g) and seed yield/plant (g) data were recorded on three randomly selected plants per plot and the average was calculated from trials at Marchouch 2014/15, Terbol 2014/15 and 2019/20. In addition, the number of pods/plant (NP) was also recorded and calculated as PH, BY and SY at Terbol 2019/20.

*The* *reduction* *indices*: The reduction index $({RI}_{trait}$) was estimated to measure the performance of selected tolerant accessions, as follows^9^:

$${RI}_{trait}=100-\frac{(100\times \overline{{\text{T}}})}{\overline{{\text{C}}}}$$

where ($\overline{{\text{T}}}$) is the trait value of evaluated accession under herbicide treatments and $\overline{{\text{C}}}$ is the value of the same accession under controlled conditions without any herbicide treatments. This reduction index was calculated for DF, DM, PH, NP, BY and SY at Terbol in 2019/20. At Marchouch in 2014/15, only the reduction indices for PH, BY and SY were calculated.

### DNA extraction and genotyping by sequencing analysis

DNA was extracted from young leaves of seedlings aged between 4 and 6 weeks, prior to the application of salt treatment, using the CTAB method, as outlined by Rogers and Bendich^77^. A total of 50 μl of 100 ng/μl DNA from each sample was sent to Agriculture Victoria, Melbourne, where Multispecies Pulse SNP chip was used for genotyping. To ensure the quality of the markers, we filtered them by call rates greater than 80%, minor allele frequency (MAF) of ≥ 5%, and heterozygosity of ≤ 15%. Only those markers that met these criteria were selected for genome-wide association analysis.

### Phenotypic data analysis

The spatial statistical row-column model was used to detect differences among genotypes (G) under different herbicide treatments (T), location (L) and their interactions (G × T), (G × L) and (G × T × L) for phenological and agronomic traits using Genstat V. 19^78^. The significance of variation among accessions and herbicide treatments was tested using p values. The best linear unbiased prediction values (BLUP) of genotypes and treatment and interactions between genotypes and treatments were also estimated by Genstat V. 19.

### Genetic diversity study

The phylogenetic data analysis was carried out using the programming language R, using the clust agglomeration method of “complete”. The similarity data matrix obtained from the SNP genotyping data was then used to construct the phylogenetic tree. To visualize the tree, we used the online tool iTOL (Interactive Tree Of Life), which allowed us to color-code the samples based on their country of origin and provided a user-friendly interface for exploring and analyzing the data (Fig. 2).

#### Single-trait GWAS

The single variate mixed linear model implemented in the software GEMMA^79,80^ was used to analyze the association between each measured phenotype in each environment with the SNP data. The model used the following equation:

$${\text{y}}=\upmu +\mathrm{X\beta }+\mathrm{I\alpha }+{\text{e}}$$

where y is a vector of the phenotypes, $\upmu$ is the intercept, X is the incidence matrix assigning individuals to genotypes, $\upbeta$ is the SNP substitution effect, I is the identity matrix, $\mathrm{\alpha }$ is a vector of random effects, and e is a vector for the residuals.

#### Meta-GWAS

Meta-GWAS analysis was performed following the method described in Bolormaa et al.^22^. Briefly, the following equation was used to calculate a chi-squared statistic ($\upchi$^2^) assuming n (number of environments per trait) degrees of freedom:

$$\chi_{i}^{2} = t_{i}{\prime} V^{ - 1} t_{i}$$

where ${{\text{t}}}_{{\text{i}}}$ represents the signed t-values for the SNP (i) in all environments, and ${{\text{V}}}^{-1}$ is the inverse of the correlation of the t-values among all environments. The following equation was used to calculate ${{\text{t}}}_{{\text{i}}}$:

$${{\text{t}}}_{{\text{i}}}=\frac{{{\text{b}}}_{{\text{i}}}}{{\text{se}}({{\text{b}}}_{{\text{i}}})}$$

where ${{\text{b}}}_{{\text{i}}}$ is the SNP effect calculated in the single-trait GWAS analysis for each environment and ${\text{se}}({{\text{b}}}_{{\text{i}}})$ is its standard error. Bonferroni correlation was used to declare significance. However, all associations with p < 0.0001 were reported in the supplementary materials as suggestive associations.

### Ethical approval

The authors confirm that the study complies with local and national regulations. The seeds were collected from the GenBank of the International Center for Agricultural Research in the Dry Areas (ICARDA) for research purposes according to the International Treaty of Plant Genetic Resources for Food and Agriculture (ITPGRFA). For the collection of seeds, all relevant permits or permissions have been obtained. The seeds flow from ICARDA GenBank at Terbol to Morocco was made following the phytosanitary regulations of both countries and using the Standard Material Transfer Agreement (SMTA) governed by ITPGRFA. The experiments were conducted at ICARDA sites at Terbol and Marchouch in accordance with National and International regulations.

### Supplementary Information

Supplementary Information.

## Data Availability

The datasets generated and analyzed during the current study are available from the corresponding authors on request.

## References

[1] Johnson CR, Thavarajah P, Payne S, Moore J, Ohm J-B (2015). Processing, cooking, and cooling affect prebiotic concentrations in lentil (*Lens culinaris*
*Medikus*). J. Food Compos. Anal..

[2] Landero JL, Beltranena E, Zijlstra RT (2012). The effect of feeding lentil on growth performance and diet nutrient digestibility in starter pigs. Anim. Feed Sci. Technol..

[3] Cokkizgin A, Shtaya MJY (2013). Lentil: Origin, cultivation techniques, utilization and advances in transformation. Agric. Sci..

[4] FAOSTAT. FAOSTAT: Statistical database. *FAOSTAT: Statistical database.* http://www.faostat.fao.org. (2023).

[5] Rubiales D, Fernández Aparicio M (2012). Innovations in parasitic weeds management in legume crops. Rev. Agron. Sustain. Dev..

[6] Elkoca E, Kantar F, Zengin H (2005). Weed control in lentil (*Lens culinaris*) in eastern Turkey. New Zeal. J. Crop Hortic. Sci..

[7] Balech R (2022). Evaluation of performance and stability of new sources for tolerance to post-emergence herbicides in lentil (*Lens culinaris* ssp. *culinaris* Medik.). Crop Pasture Sci..

[8] Sharma SR, Singh S, Aggarwal N, Kushwah A, Kumar S (2017). Inherent variability among different lentil (*Lens culinaris* medik.) genotypes against tolerance to metribuzin herbicide. Biochem. Cell. Arch..

[9] Sharma SR (2018). Genetic variation for tolerance to post-emergence herbicide, imazethapyr in lentil (*Lens culinaris* Medik.). Arch. Agron. Soil Sci..

[10] Abou-Khater L (2021). Identification of tolerance to metribuzin and Imazethapyr herbicides in faba bean (*Vicia*
*faba* L.). Crop Sci..

[11] Gaur P (2013). Large genetic variability in chickpea for tolerance to herbicides imazethapyr and metribuzin. Agronomy.

[12] Stewart CL, Nurse RE, Hamill AS, Sikkema PH (2010). Environment and soil conditions influence pre-and postemergence herbicide efficacy in soybean. Weed Technol..

[13] Kumar S, Rajendran K, Kumar J, Hamwieh A, Baum M (2015). Current knowledge in lentil genomics and its application for crop improvement. Front. Plant Sci..

[14] Arumuganathan K, Earle ED (1991). Nuclear DNA content of some important plant species. Plant Mol. Biol. Rep..

[15] Bett, K. The lentil genome–from the sequencer to the field. (2016).

[16] Khazaei H (2017). Marker–trait association analysis of iron and zinc concentration in lentil (*Lens*
*culinaris*
*Medik*) seeds. Plant Genome.

[17] Sudheesh S (2016). SNP-based linkage mapping for validation of QTLs for resistance to *Ascochyta*
*blight* in lentil. Front. Plant Sci..

[18] Singh A, Dikshit HK, Mishra GP, Aski M, Kumar S (2019). Association mapping for grain diameter and weight in lentil using SSR markers. Plant Genet..

[19] Verma P (2015). Construction of a genetic linkage map and identification of QTLs for seed weight and seed size traits in lentil (*Lens*
*culinaris*
*M*edik.). PLoS ONE.

[20] Merrick LF, Burke AB, Zhang Z, Carter AH (2022). Comparison of single-trait and multi-trait genome-wide association models and inclusion of correlated traits in the dissection of the genetic architecture of a complex trait in a breeding program. Front. Plant Sci..

[21] Evangelou E, Ioannidis JP (2013). Meta-analysis methods for genome-wide association studies and beyond. Nat. Rev. Genet..

[22] Bolormaa S (2014). A multi-trait, meta-analysis for detecting pleiotropic polymorphisms for stature, fatness and reproduction in beef cattle. PLoS Genet..

[23] Joukhadar R, Daetwyler HD, Joukhadar R, Daetwyler HD (2022). Data integration, imputation imputation, and meta-analysis meta-analysis for genome-wide association studies. Genome-Wide Association Studies.

[24] Balech, R. *et al.* Identification of novel genes associated with herbicide tolerance in Lentil (*Lens culinaris* spp. *culinaris* Medik.). In *International Conference on, ‘Pulses: Smart Crops for Agricultural Sustainability and Nutritional Security’.* 441 (2023). Accessed 4 July 2017.

[25] Shoup DE, Al-Khatib K, Peterson DE (2003). Common waterhemp (*Amaranthus rudis*) resistance to protoporphyrinogen oxidase-inhibiting herbicides. Weed Sci..

[26] Taran B, Warkentin TD, Vandenberg A, Holm FA (2010). Variation in chickpea germplasm for tolerance to imazethapyr and imazamox herbicides. Can. J. Plant Sci..

[27] Goud VV, Murade NB, Khakre MS, Patil AAN (2013). Efficacy of imazethapyr and quizalofop-ethil herbicides on growth and yield of chickpea. Bioscan.

[28] Ma Y (2020). Dissecting the genetic architecture of *Aphanomyces* root rot resistance in lentil by QTL mapping and genome-wide association study. Int. J. Mol. Sci..

[29] Johnson N (2021). Genome-wide association mapping of lentil (*Lens culinaris*
*Medikus*) prebiotic carbohydrates toward improved human health and crop stress tolerance. Sci. Rep..

[30] Gela T, Ramsay L, Haile TA, Vandenberg A, Bett K (2021). Identification of anthracnose race 1 resistance loci in lentil by integrating linkage mapping and genome-wide association study. Plant Genome.

[31] Henares BM (2023). Virulence profiles and genome-wide association study for *Ascochyta*
*lentis* isolates collected from Australian lentil-growing regions. Phytopathology.

[32] Hang J, Shi D, Neufeld J, Bett KE, House JD (2022). Prediction of protein and amino acid contents in whole and ground lentils using near-infrared reflectance spectroscopy. LWT.

[33] Kumar J, Gupta D, SenBaum M, Varshney RK, Kumar S (2021). Genomics-assisted lentil breeding: Current status and future strategies. Legum. Sci..

[34] Winkler TW (2014). Quality control and conduct of genome-wide association meta-analyses. Nat. Protoc..

[35] Joukhadar R (2021). Meta-analysis of genome-wide association studies reveal common loci controlling agronomic and quality traits in a wide range of normal and heat stressed environments. Theor. Appl. Genet..

[36] Singh J (2021). Identifying and validating SSR markers linked with rust resistance in lentil (*Lens culinaris*). Plant Breed..

[37] Zhang J (2015). Identification of putative candidate genes for water stress tolerance in canola (*Brassica napus*). Front. Plant Sci..

[38] Coser SM (2017). Genetic architecture of charcoal rot (*Macrophomina phaseolina*) resistance in soybean revealed using a diverse panel. Front. Plant Sci..

[39] Moellers TC (2017). Main and epistatic loci studies in soybean for *Sclerotinia*
*sclerotiorum* resistance reveal multiple modes of resistance in multi-environments. Sci. Rep..

[40] Chen G (2010). Identification of QTL for oil content, seed yield, and flowering time in oilseed rape (*Brassica napus*). Euphytica.

[41] Shook JM (2021). Meta-GWAS for quantitative trait loci identification in soybean. G3 Genes Genom. Genet..

[42] Battenfield SD (2018). Breeding-assisted genomics: Applying meta-GWAS for milling and baking quality in CIMMYT wheat breeding program. PLoS ONE.

[43] Fikere M (2020). Meta-analysis of GWAS in canola blackleg (*Leptosphaeria maculans*) disease traits demonstrates increased power from imputed whole-genome sequence. Sci. Rep..

[44] Lagunas B (2019). Regulation of resource partitioning coordinates nitrogen and rhizobia responses and autoregulation of nodulation in *Medicago truncatula*. Mol. Plant.

[45] Pang S (2012). Co-induction of a glutathione-S-transferase, a glutathione transporter and an ABC transporter in maize by xenobiotics. PLoS ONE.

[46] Stacey G, Koh S, Granger C, Becker JM (2002). Peptide transport in plants. Trends Plant Sci..

[47] Sánchez-Fernández R, Davies TE, Coleman JO, Rea PA (2001). The *Arabidopsis thaliana* ABC protein superfamily, a complete inventory. J. Biol. Chem..

[48] Song WY (2014). A rice ABC transporter, OsABCC1, reduces arsenic accumulation in the grain. Proc. Nat. Acad. Sci..

[49] Yazaki K, Shitan N, Sugiyama A, Takanashi K (2009). Cell and molecular biology of ATP-binding cassette proteins in plants. Int. Rev. Cell Mol. Biol..

[50] Van Eerd LL, Hoagland RE, Zablotowicz RM, Hall JC (2003). Pesticide metabolism in plants and microorganisms. Weed Sci..

[51] Kurya B, Mia MS, Liu H, Yan G (2022). Genomic regions, molecular markers, and flanking genes of metribuzin tolerance in wheat (*Triticum*
*aestivum* L.).. Front. Plant Sci..

[52] Bhoite R, Si P, Siddique KH, Yan G (2021). Comparative transcriptome analyses for metribuzin tolerance provide insights into key genes and mechanisms restoring photosynthetic efficiency in bread wheat (*Triticum aestivum* L.). Genomics.

[53] Manabe Y, Tinker N, Colville A, Miki B (2007). CSR1, the sole target of imidazolinone herbicide in *Arabidopsis thaliana*. Plant Cell Physiol..

[54] Abusteit EO (1985). Absorption, translocation, and metabolism of metribuzin in diploid and tetraploid soybean (*Glycine max*) plants and cell cultures. Weed Sci..

[55] Kim GJ, Kim HS (1998). Identification of the structural similarity in the functionally related amidohydrolases acting on the cyclic amide ring. Biochem. J..

[56] Moffatt, B. A., Ashihara, H. Purine and pyrimidine nucleotide synthesis and metabolism. *The Arabidopsis Book/American Society of Plant Biologists* 1 (2002). 10.1199/tab.0018 PMC3243375 22303196

[57] Dong Q (2019). UMP kinase regulates chloroplast development and cold response in rice. Int. J. Mol. Sci..

[58] Hopfinger AJ (1980). A QSAR investigation of dihydrofolate reductase inhibition by Baker triazines based upon molecular shape analysis. J. Am. Chem. Soc..

[59] Duran, V. The Role of Allantoinase in Soybean (*Glycine**max* L.) Plants (Doctoral dissertation). (2012).

[60] Kafer CW (2002). Characterization of the De Novo Pyrimidine Biosynthetic Pathway in Arabidopsis Thaliana.

[61] Wang Y (2023). Multi-omics analysis reveals the regulatory and metabolic mechanisms underlying low-nitrogen tolerance at the flowering stage in rice. Agronomy.

[62] Baud S (2003). Multifunctional acetyl-CoA carboxylase 1 is essential for very long chain fatty acid elongation and embryo development in Arabidopsis. Plant J..

[63] Capron, A., Chatfield, S., Provart, N. & Berleth, T. Embryogenesis: Pattern formation from a single cell. *The Arabidopsis Book.* 7 (2009). 10.1199/tab.0126 PMC3243344 22303250

[64] Chen J, Song Y, Zhang H, Zhang D (2013). Genome-wide analysis of gene expression in response to drought stress in *Populus simonii*. Plant Mol. Biol. Rep..

[65] Bharadwaj B (2023). Physiological and genetic responses of lentil (*Lens culinaris*) under flood stress. Plant Stress.

[66] Elborough KM (1996). Biotin carboxyl carrier protein and carboxyltransferase subunits of the multi-subunit form of acetyl-CoA carboxylase from *Brassica napus*: Cloning and analysis of expression during oilseed rape embryogenesis. Biochem. J..

[67] Megha S, Wang Z, Kav NN, Rahman H (2022). Genome-wide identification of biotin carboxyl carrier subunits of acetyl-CoA carboxylase in Brassica and their role in stress tolerance in oilseed *Brassica napus*. BMC Genom..

[68] Xie Q (2021). Abscisic acid regulates the root growth trajectory by reducing auxin transporter PIN2 protein levels in *Arabidopsis thaliana*. Front. Plant Sci..

[69] Madoka Y (2002). Chloroplast transformation with modified accD operon increases acetyl-CoA carboxylase and causes extension of leaf longevity and increase in seed yield in tobacco. Plant Cell Physiol..

[70] Wu JH (2021). A myeloid leukemia factor homolog involved in encystation-induced protein metabolism in *Giardia*
*lamblia* biochim. Biophys. Acta. BBA Gen. Subj..

[71] Wang S, de Vries SC (2013). Toxicogenomics-Based In Vitro Alternatives for Estrogenicity Testing.

[72] Arce AL, Cabello JV, Chan RL (2008). Patents on plant transcription factors. Recent Pat. Biotechnol..

[73] Burke R (2020). Stressed to death: The role of transcription factors in plant programmed cell death induced by abiotic and biotic stimuli. Front. Plant Sci..

[74] Radwan DEM (2012). Salicylic acid induced alleviation of oxidative stress caused by clethodim in maize (*Zea mays* L.) leaves. Pestic. Biochem. Physiol..

[75] Tausz, M. The role of glutathione in plant response and adaptation to natural stress. In *Significance of Glutathione to Plant Adaptation to the Environment.* 101–122 (2001).

[76] Kumar, S. & Rajendran, K. *Lentil Ontology*. https://cropontology.org/term/CO_339:ROOT. (2016).

[77] Rogers SO, Bendich AJ (1985). Extraction of DNA from milligram amounts of fresh, herbarium and mummified plant tissues. Plant Mol. Biol..

[78] Goedhart, P. W. & Thissen, J. T. N. M. *Biometris GenStat Procedure Library Manual*. (2018).

[79] Zhou X, Stephens M (2014). Efficient multivariate linear mixed model algorithms for genome-wide association studies. Nat. Methods.

[80] Zhou X, Stephens M (2012). Genome-wide efficient mixed-model analysis for association studies. Nat. Genet..

