# Supplementary material for: Identification of novel genes associated with herbicide tolerance in Lentil (Lens culinaris ssp. culinaris Medik.)
Source: Sci Rep. 2024 May 3;14:10215. doi: 10.1038/s41598-024-59695-z (PMC11068770; doi:10.1038/s41598-024-59695-z)
Supplement: Supplementary file 1 — Supplementary Information. [file 41598_2024_59695_MOESM1_ESM.pdf]

Identification of novel genes associated with herbicide tolerance in Lentil (Lens culinaris ssp. culinaris Medik.).

Authors: Rind Balech[1*], Fouad Maalouf[1*], Sukhjiwan Kaur[2] Abdulqader Jighly, Reem Joukhadar, Alsamman M. Alsamman[3], Aladdin Hamwieh[3], Lynn Abou Khater[1], Diego Rubiales[4] and Shiv Kumar[5]

[1] International Center for Agricultural Research in the Dry Areas (ICARDA), Terbol, Lebanon.
[2] AgriBio, Centre for AgriBioscience, Department of Energy, Environment and Climate Action, 5 Ring Road, Bundoora, Victoria 3083, Australia.
[3] ICARDA, Cairo, Egypt.
[4] Institute for Sustainable Agriculture, CSIC, Córdoba, Spain.
[5] ICARDA, New Delhi, India.
*Contact email: r.balech@cgiar.org, f.maalouf@cgiar.org

Supplementary Table S1: List of lentil accessions evaluated for Imazethapyr and Metribuzin tolerance at Terbol and Marchouch.

SN	Line	IG	CROP_NO	Origin
**1**	ILL98	98	98	Morocco
**2**	ILL129	129	129	Turkey
**3**	ILL191	191	191	Lebanon
**4**	ILL195	195	195	Turkey
**5**	ILL213	213	213	Afghanistan
**6**	ILL223	223	223	Iran
**7**	ILL257	257	257	Iran
**8**	ILL262	262	262	Cyprus
**9**	ILL304	304	304	Greece
**10**	ILL323	323	323	Serbia
**11**	ILL348	348	348	Mexico
**12**	ILL358	358	358	Mexico
**13**	ILL361	361	361	Chile
**14**	ILL459	459	459	Chile
**15**	ILL461	461	461	Chile
**16**	ILL494	494	494	Guatemala
**17**	ILL502	502	502	Mexico
**18**	ILL556	556	556	Turkey
**19**	ILL590	590	590	Turkey
**20**	ILL595	595	595	Ukraine
**21**	ILL597	597	597	Russia
**22**	ILL624	624	624	Macedonia
**23**	ILL705	705	705	Poland
**24**	ILL719	719	719	Hungary
**25**	ILL769	769	769	Iran

**26**	ILL840	840	840	Lebanon
**27**	ILL857	857	857	Alegria
**28**	ILL890	890	890	Cyprus
**29**	ILL918	918	918	Tunisia
**30**	ILL931	931	931	India
**31**	ILL950	950	950	Yemen
**32**	ILL956	956	956	Chile
**33**	ILL960	960	960	Iran
**34**	ILL975	975	975	Chile
**35**	ILL1005	1005	1005	Chile
**36**	ILL1455	1455	1455	Iran
**37**	ILL1460	1460	1460	Iran
**38**	ILL1878	1878	1878	Turkey
**39**	ILL1918	1918	1918	Portugual
**40**	ILL1939	1939	1939	Morocco
**41**	ILL1959	1959	1959	Ethiopia
**42**	ILL2131	2131	2131	Syria
**43**	ILL2181	2181	2181	Turkey
**44**	ILL2194	2194	2194	Pakistan
**45**	ILL2230	2230	2230	Yugoslavia
**46**	ILL2297	2297	2297	Pakistan
**47**	ILL2442	2442	2442	Afghanistan
**48**	ILL2445	2445	2445	Afghanistan
**49**	ILL3485	3485	3485	Nepal
**50**	ILL3487	3487	3487	Nepal
**51**	ILL3517	3517	3517	India
**52**	ILL4152	4152	4152	India
**53**	ILL4164	4164	4164	India
**54**	ILL4401	4401	4401	Syria
**55**	ILL4471	4471	4471	Syria
**56**	ILL4606	4606	4606	Palestine
**57**	ILL4637	4637	4637	Chile
**58**	ILL4671	4671	4671	United States of America
**59**	ILL4774	4774	4774	Romania
**60**	ILL4781	4781	4781	Alegria
**61**	ILL4782	4782	4782	Norway
**62**	ILL4791	4791	4791	Iran
**63**	ILL4804	4804	4804	Libya
**64**	ILL4819	4819	4819	Russia
**65**	ILL4830	4830	4830	Russia
**66**	ILL4831	4831	4831	Germany
**67**	ILL4841	4841	4841	Albania
**68**	ILL4857	4857	4857	Greece

**69**	ILL4881	4881	4881	Germany
**70**	ILL4886	4886	4886	Iran
**71**	ILL4915	4915	4915	Croatia
**72**	ILL4956	4956	4956	Portugal
**73**	ILL5028	5028	5028	Spain
**74**	ILL5151	5151	5151	India
**75**	ILL5244	5244	5244	Jordan
**76**	ILL5416	5416	5416	Italy
**77**	ILL5424	5424	5424	Poland
**78**	ILL5480	5480	5480	Czechia
**79**	ILL5505	5505	5505	Sudan
**80**	ILL5509	5509	5509	Syria
**81**	ILL5533	5533	5533	Greece
**82**	ILL5553	5553	5553	Mexico
**83**	ILL5588	5588	5588	Jordan
**84**	ILL5595	5595	5595	Syria
**85**	ILL5626	5626	5626	Lebanon
**86**	ILL5628	5628	5628	Spain
**87**	ILL5645	5645	5645	Mexico
**88**	ILL5722	5722	5722	ICARDA
**89**	ILL5769	5769	5769	ICARDA
**90**	ILL5883	69492	5883	Jordan
**91**	ILL5968	69577	5968	Cyprus
**92**	ILL6015	70070	6015	ICARDA
**93**	ILL6021	70076	6021	Syria
**94**	ILL6025	70080	6025	Syria
**95**	ILL6126	70181	6126	Tajikistan
**96**	ILL6207	71139	6207	ICARDA
**97**	ILL6258	71190	6258	ICARDA
**98**	ILL6434	71366	6434	Syria
**99**	ILL6447	71379	6447	ICARDA
**100**	ILL6467	71399	6467	ICARDA
**101**	ILL6644	71595	6644	Syria
**102**	ILL6750	71701	6750	Syria
**103**	ILL6778	73642	6778	Syria
**104**	ILL6783	73647	6783	ICARDA
**105**	ILL6789	73653	6789	Syria
**106**	ILL6811	73675	6811	ICARDA
**107**	ILL6848	73712	6848	Syria
**108**	ILL6870	73734	6870	Syria
**109**	ILL6925	73789	6925	Jordan
**110**	ILL6991	73855	6991	ICARDA
**111**	ILL6994	73858	6994	ICARDA

**112**	ILL7010	73874	7010	ICARDA
**113**	ILL7070	73934	7070	Syria
**114**	ILL7084	73948	7084	Italy
**115**	ILL7115	73979	7115	United States of America
**116**	ILL7127	75846	7127	Syria
**117**	ILL7162		7162	ICARDA
**118**	ILL7163	75882	7163	Pakistan
**119**	ILL7201	75920	7201	ICARDA
**120**	ILL7210	75929	7210	ICARDA
**121**	ILL7213	75932	7213	Syria
**122**	ILL7437	76156	7437	Nepal
**123**	ILL7502	76221	7502	ICARDA
**124**	ILL7532	76251	7532	ICARDA
**125**	ILL7537	76256	7537	ICARDA
**126**	ILL7547	76266	7547	ICARDA
**127**	ILL7617	109076	7617	ICARDA
**128**	ILL7620	109079	7620	ICARDA
**129**	ILL7644	108550	7644	Pakistan
**130**	ILL7650	108568	7650	Pakistan
**131**	ILL7661	114663	7661	ICARDA
**132**	ILL7666	114668	7666	ICARDA
**133**	ILL7668	114670	7668	ICARDA
**134**	ILL7670	114672	7670	ICARDA
**135**	ILL7685	114687	7685	ICARDA
**136**	ILL7686	114688	7686	ICARDA
**137**	ILL7698	114700	7698	ICARDA
**138**	ILL7706	114708	7706	ICARDA
**139**	ILL7711	114713	7711	ICARDA
**140**	ILL7716	114718	7716	ICARDA
**141**	ILL7726	111986	7726	Morocco
**142**	ILL7727	111991	7727	Morocco
**143**	ILL7745	113079	7745	Saudi Arabia
**144**	ILL7915	115370	7915	Nepal
**145**	ILL7935	117635	7935	ICARDA
**146**	ILL7946	117646	7946	ICARDA
**147**	ILL7959	117659	7959	ICARDA
**148**	ILL7978	117678	7978	ICARDA
**149**	ILL7979	117679	7979	ICARDA
**150**	ILL7981	117681	7981	ICARDA
**151**	ILL7983	117683	7983	ICARDA
**152**	ILL7984	117684	7984	ICARDA
**153**	ILL7989	117689	7989	ICARDA
**154**	ILL7990	117690	7990	ICARDA

**155**	ILL8006	75499	8006	Syria
**156**	ILL8008			ICARDA
**157**	ILL8009			ICARDA
**158**	ILL8070	122882	8070	ICARDA
**159**	ILL8077	122889	8077	ICARDA
**160**	ILL8090	122902	8090	ICARDA
**161**	ILL8095	122907	8095	ICARDA
**162**	ILL8109	122915	8109	Argentina
**163**	ILL8110	122916	8110	Bulgaria
**164**	ILL8112	122918	8112	Pakistan
**165**	ILL8115	122921	8115	ICARDA
**166**	ILL8128	122934	8128	ICARDA
**167**	ILL8194	124523	8194	ICARDA
**168**	ILL8195	124524	8195	ICARDA
**169**	ILL8595	123846	8595	Ecuador
**170**	ILL8614	129136	8614	ICARDA
**171**	ILL8620	129142	8620	ICARDA
**172**	ILL8632	129163	8632	ICARDA
**173**	ILL9850	137959	9850	ICARDA
**174**	ILL9882	137991	9882	ICARDA
**175**	ILL9951	138060	9951	ICARDA
**176**	ILL9997	138106	9997	ICARDA
**177**	ILL10657	143547	10657	Turkey
**178**	ILL10712	156536	10712	ICARDA
**179**	ILL10713	156537	10713	ICARDA
**180**	ILL10737	156560	10737	ICARDA
**181**	ILL10738	156561	10738	ICARDA
**182**	ILL10740	156563	10740	ICARDA
**183**	ILL10742	156565	10742	ICARDA
**184**	ILL10748	156571	10748	ICARDA
**185**	ILL10756	156579	10756	ICARDA
**186**	ILL10758	156581	10758	ICARDA
**187**	ILL10810	156633	10810	ICARDA
**188**	ILL10812	156635	10812	ICARDA
**189**	ILL10816	156639	10816	ICARDA
**190**	ILL10825	156648	10825	ICARDA
**191**	ILL10826	156649	10826	ICARDA
**192**	ILL10827	156650	10827	ICARDA
**193**	ILL10833	156656	10833	ICARDA
**194**	ILL10870	156693	10870	Ethiopia
**195**	ILL10903	156726	10903	ICARDA
**196**	ILL10907	156730	10907	ICARDA
**197**	ILL10912	156735	10912	ICARDA

**198**	ILL10924	156747	10924	Australia
**199**	ILL10946	156769	10946	Australia
**200**	ILL10948	156771	10948	ICARDA
**201**	ILL10967	156790	10967	ICARDA
**202**	ILL8113	122919	PAK91517	ICARDA
**203**	010S 96130-1	76175		Lebanon
**204**	010S 96155-2	76185		Lebanon
**205**	06S 53110-02	76179		Lebanon
**206**	06S 53110-03	76188		Lebanon
**207**	08S 40111-01	76190		Lebanon
**208**	2009S 96102-7			ICARDA
**209**	2009S 96573-3			ICARDA
**210**	Aljabl	916	916	India
**211**	GUDO	5748	5748	ICARDA
**212**	L24			ICARDA
**213**	LC99602614Z			ICARDA
**214**	LRIL-21-111	157234	11411	Bangladesh
**215**	LRIL-22-46	157239	11416	ICARDA
**216**	Nipal-CB-20-1			ICARDA
**217**	Nipal-CB-38-2			ICARDA
**218**	NIPAL-CB-52-2			ICARDA
**219**	Nipal-CB-83-1			ICARDA
**220**	PAK 88527	122920	8114	ICARDA
**221**	USA-3			ICARDA
**222**	YL 350020			ICARDA
**223**	4400	4400	4400	Syria
**224**	4605	4605	4605	Argentina
**225**	ILL174	174	174	Turkey
**226**	ILL353	353	353	Mexico
**227**	ILL468	468	468	Chile
**228**	ILL5488	5488	5488	Chile
**229**	ILL2126	2126	2126	Syria
**230**	ILL2439	2439	2439	Ethiopia
**231**	ILL2580	2580	2580	India
**232**	ILL5562	5562	5562	Jordan
**233**	ILL5582	5582	5582	Jordan
**234**	GUDO	5748	5748	ICARDA
**235**	ILL5888	69497	5888	Bangladesh
**236**	ILL6004	70059	6004	ICARDA
**237**	ILL6024	70079	6024	ICARDA
**238**	ILL6037	70092	6037	ICARDA
**239**	ILL6199	71131	6199	ICARDA
**240**	ILL6204	71136	6204	ICARDA

**241**	ILL6212	71144	6212	ICARDA
**242**	ILL6243	71175	6243	ICARDA
**243**	ILL6346	71278	6346	Pakistan
**244**	ILL6359	71291	6359	Pakistan
**245**	ILL6816	73680	6816	ICARDA
**246**	ILL7012	73876	7012	ICARDA
**247**	ILL7250	75969	7250	Nepal
**248**	ILL7308	76027	7308	Nepal
**249**	ILL7543	76262	7543	ICARDA
**250**	ILL7553	76272	7553	Turkey
**251**	ILL7683	114685	7683	ICARDA
**252**	ILL7950	117650	7950	ICARDA
**253**	ILL8010			ICARDA
**254**	ILL8018	117711	8018	Pakistan
**255**	ILL8066	122878	8066	ICARDA
**256**	ILL8068	122880	8068	ICARDA
**257**	ILL8072	122884	8072	ICARDA
**258**	ILL8078	122890	8078	ICARDA
**259**	ILL8089	122901	8089	ICARDA
**260**	ILL8111	122917	8111	Bulgaria
**261**	ILL8190	124519	8190	ICARDA
**262**	ILL8610	129132	8610	ICARDA
**263**	ILL9945	138054	9945	ICARDA
**264**	ILL623	623	623	Macedonia
**265**	ILL1097	1097	1097	Iran
**266**	ILL2406	2406	2406	Iran
**267**	ILL2684	2684	2684	India
**268**	ILL6075	70130	6075	Pakistan
**269**	ILL7804	114892	7804	Nepal
**270**	ILL7814	114931	7814	Nepal
**271**	ILL7820	114951	7820	Nepal
**272**	ILL7833	115006	7833	Nepal
**273**	ILL10690	156514	10690	Australia
**274**	ILL10864	156687	10864	ICARDA
**275**	ILL82	82	82	Ukraine
**276**	ILL158	158	158	Syria
**277**	ILL224	224	224	Belgium
**278**	ILL268	268	268	Argentina
**279**	ILL490	490	490	Syria
**280**	ILL619	619	619	Armenia
**281**	ILL858	858	858	Alegria
**282**	ILL1013	1013	1013	Iran
**283**	ILL5988	70043	5988	ICARDA

**284**	ILL1861	1861	1861	Sudan
**285**	ILL1949	1949	1949	Serbia
**286**	ILL4409	4409	4409	Czechia
**287**	ILL4738	4738	4738	Canada
**288**	ILL4900	4900	4900	India
**289**	ILL4926	4926	4926	Spain
**290**	ILL5261	5261	5261	Jordan
**291**	ILL5653	5653	5653	Spain
**292**	ILL6350	71282	6350	Pakistan

Supplementary Figure S2: (A): Precipitation(mm) trends during the cropping seasons of the screened years at Terbol and Marchouch. (B): Variation of maximum temperature (˚C) during the different cropping seasons. (C): Minimum temperature (˚C) during the different cropping seasons.

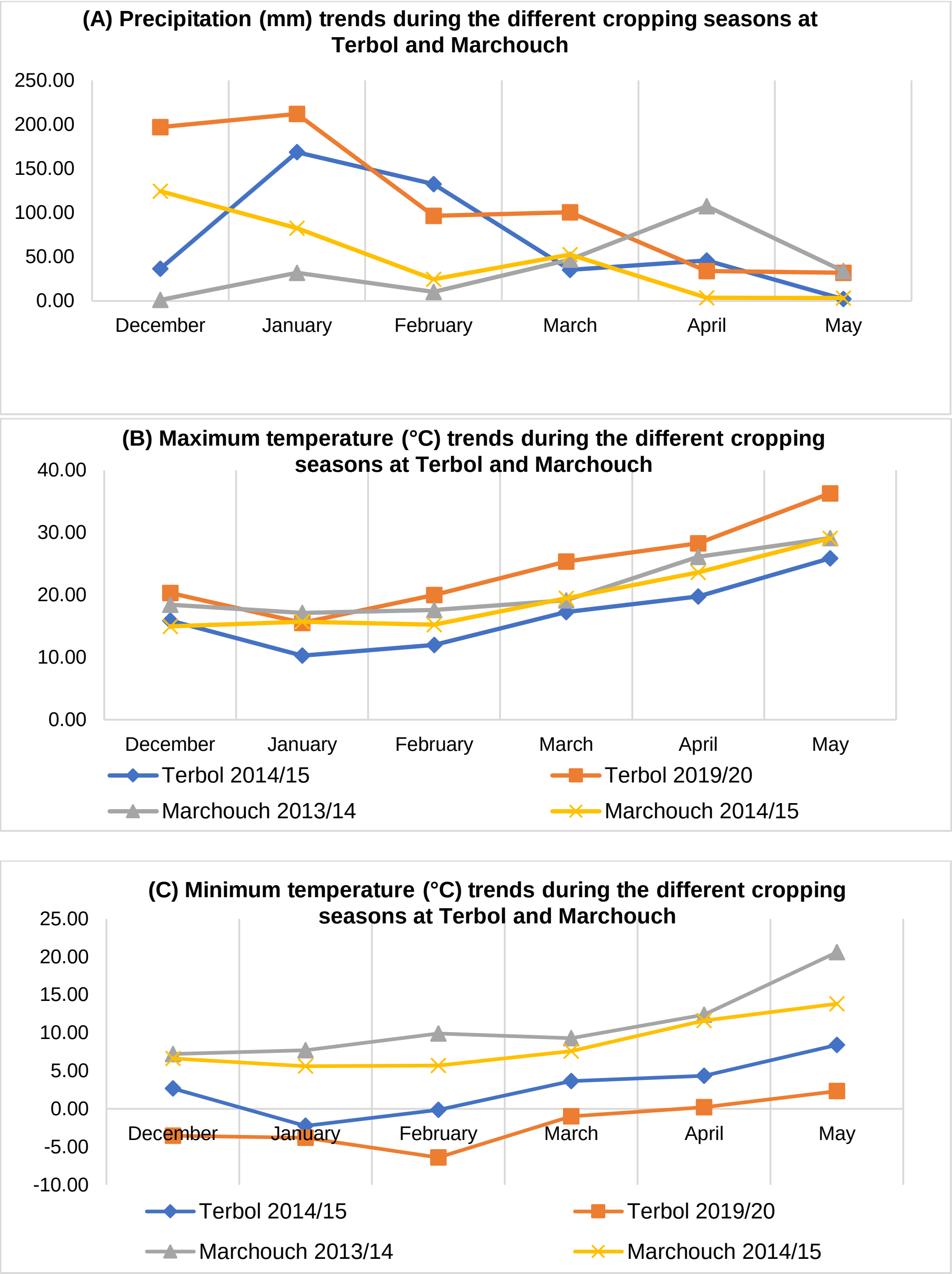

Supplementary Table S3: SNP-trait associations revealed by GWAS analysis; bolded (-Log10) values of SNPs represents the highly significant associations.

QTL	SNP	Chr	allele1	allele0	MAF	HDS2	DFLR	RI_{DFLR}	DMAT	RI_{DMAT}	PH	BY	SY	NPP	R_{INPP}	NSP
**QTL001**	AVR-Lc-00233.01-022617381	1	C	T	0.062	1.0	0.1	0.4	5.1	0.1	0.1	0.3	0.1	0.7	0.7	0.5
**QTL004**	AVR-Lc-00525.01-355542407	1	G	T	0.31	2.0	0.3	0.4	4.1	0.6	0.2	2.2	3.0	0.6	0.8	0.4
**QTL004**	AVR-Lc-00526.01-355768105	1	G	A	0.306	0.0	1.1	0.1	4.6	0.2	0.3	0.8	0.5	0.3	0.4	1.1
**QTL005**	AVR-Lc-00556.01-362542743	1	T	C	0.242	1.0	2.3	0.5	4.5	0.6	0.1	1.9	1.8	0.3	0.4	0.3
**QTL006**	AVR-Lc-00578.01-366320966	1	C	T	0.253	0.8	0.0	0.1	1.5	0.1	0.0	1.7	0.1	2.1	4.5	2.4
**QTL006**	AVR-Lc-00579.01-366322027	1	A	G	0.136	0.5	0.2	0.1	0.9	0.0	0.1	0.7	0.1	1.0	**5.7**	0.8
**QTL007**	AVR-Lc-00831.01-430032156	1	T	C	0.051	0.2	0.2	2.2	0.0	0.3	1.0	0.3	0.2	4.6	0.1	2.1
**QTL007**	AVR-Lc-00835.01-430931278	1	A	G	0.062	0.1	0.6	1.5	0.2	1.1	0.1	2.5	0.4	**6.0**	0.0	2.9
**QTL007**	AVR-Lc-00836.01-431094892	1	C	T	0.071	0.2	0.8	0.5	0.2	0.4	0.5	1.2	0.3	4.1	0.1	1.5
**QTL002**	AVR-Lc-00911.01-044349219	1	T	C	0.335	1.0	0.9	4.4	0.9	0.3	0.6	0.7	1.3	0.1	0.3	0.4
**QTL008**	AVR-Lc-00928.01-446895597	1	A	G	0.115	0.0	0.8	0.7	0.3	1.8	0.9	0.6	4.9	0.5	0.0	0.7
**QTL009**	AVR-Lc-01006.01-468700401	1	C	T	0.111	0.1	1.5	0.4	1.5	0.0	0.4	0.3	0.7	1.9	0.0	4.4
**QTL010**	AVR-Lc-01313.01-530708051	1	C	T	0.058	0.2	0.0	0.1	1.0	0.0	0.0	0.0	0.7	0.1	4.6	0.0
**QTL011**	AVR-Lc-01352.01-535793448	1	G	A	0.142	0.5	0.6	0.7	0.6	0.2	0.6	0.5	1.1	0.7	**6.8**	0.1
**QTL012**	AVR-Lc-01367.01-537371482	1	C	A	0.077	0.0	**6.5**	1.7	1.5	1.4	2.2	0.1	0.3	0.0	0.1	0.3
**QTL003**	AVR-Lc-01375.01-054201950	1	G	A	0.054	1.4	0.6	0.3	0.4	0.3	0.3	1.7	0.4	4.5	0.2	3.7
**QTL015**	AVR-Lc-01558.02-012643639	2	T	C	0.114	0.5	3.1	4.0	0.1	0.0	0.5	0.7	0.2	0.8	0.3	0.6
**QTL016**	AVR-Lc-01636.02-014499828	2	C	T	0.383	4.2	0.4	0.9	0.6	0.2	0.9	1.0	0.1	1.7	0.5	0.9
**QTL013**	AVR-Lc-01885.02-000213238	2	C	T	0.409	**6.3**	0.8	1.2	0.5	0.1	0.7	1.6	0.4	2.4	0.6	1.0
**QTL017**	AVR-Lc-02107.02-281092190	2	C	T	0.197	4.8	0.1	0.6	0.2	0.2	0.1	1.2	0.2	1.5	0.3	0.8

**QTL018**	AVR-Lc-02134.02-290328617	2	C	A	0.075	0.8	0.3	4.2	0.6	0.0	0.2	0.2	0.5	0.5	0.3	0.8
**QTL019**	AVR-Lc-02169.02-302897953	2	A	G	0.134	0.1	0.1	0.6	0.3	4.6	0.1	0.0	0.1	0.0	0.1	0.3
**QTL019**	AVR-Lc-02189.02-307011079	2	A	G	0.104	0.1	0.3	0.1	1.0	**9.1**	1.1	0.1	0.1	0.0	0.0	0.3
**QTL019**	AVR-Lc-02200.02-309350505	2	C	T	0.13	0.2	0.3	0.3	1.0	**6.3**	1.4	0.4	0.5	0.0	0.1	0.0
**QTL020**	AVR-Lc-02445.02-370911708	2	G	A	0.078	0.7	0.3	5.0	1.0	0.0	0.3	0.5	0.7	0.8	0.1	0.7
**QTL020**	AVR-Lc-02544.02-393755630	2	C	T	0.134	0.2	0.2	4.6	0.3	0.4	0.3	0.3	0.3	0.2	0.8	1.0
**QTL021**	AVR-Lc-02714.02-436766259	2	G	A	0.071	0.4	1.0	0.2	0.8	0.5	0.4	**5.6**	1.3	2.7	0.1	1.5
**QTL021**	AVR-Lc-02715.02-436994699	2	G	A	0.084	0.8	0.5	0.3	0.5	0.3	0.8	**5.9**	1.6	3.4	0.0	2.8
**QTL022**	AVR-Lc-02719.02-437786468	2	T	G	0.395	2.0	0.7	2.0	4.0	0.1	0.3	0.7	0.5	0.2	2.6	0.2
**QTL023**	AVR-Lc-02723.02-439149768	2	A	C	0.21	3.4	1.1	4.6	1.1	0.3	0.0	1.5	0.8	2.1	3.3	2.6
**QTL023**	AVR-Lc-02725.02-439730005	2	T	G	0.378	2.3	2.2	4.7	2.0	0.8	0.0	0.7	0.3	2.0	2.0	2.4
**QTL024**	AVR-Lc-02786.02-456774445	2	A	G	0.089	0.9	1.8	4.6	0.3	0.8	0.5	0.1	0.6	1.0	4.1	0.1
**QTL025**	AVR-Lc-02857.02-473502035	2	A	G	0.133	0.6	2.0	0.6	1.9	0.9	0.3	1.1	0.3	5.2	0.1	**5.5**
**QTL026**	AVR-Lc-02988.02-518425731	2	G	T	0.1	0.7	**6.2**	3.1	0.6	0.2	0.4	0.0	0.9	0.2	0.0	0.5
**QTL027**	AVR-Lc-03032.02-530710201	2	C	T	0.057	0.1	4.7	0.5	0.0	0.1	0.4	0.1	0.3	0.1	0.0	0.1
**QTL028**	AVR-Lc-03076.02-539961420	2	G	A	0.246	0.3	3.1	0.9	4.5	0.6	0.4	0.5	0.9	1.3	0.3	0.4
**QTL029**	AVR-Lc-03296.02-599856144	2	T	G	0.089	0.7	1.2	1.3	0.2	0.9	0.5	1.5	0.7	**5.7**	0.0	4.5
**QTL030**	AVR-Lc-03341.02-605719692	2	G	A	0.351	0.4	0.2	4.6	0.4	1.7	0.1	0.1	0.5	0.5	0.1	1.2
**QTL031**	AVR-Lc-03373.02-608709301	2	A	G	0.092	0.6	0.7	1.2	0.6	0.3	0.2	0.1	0.1	0.6	**6.2**	0.6
**QTL032**	AVR-Lc-03379.02-609257610	2	T	C	0.085	0.1	2.5	**7.1**	0.5	0.9	2.2	0.0	1.7	0.0	0.0	0.5
**QTL014**	AVR-Lc-03458.02-007762915	2	C	T	0.05	0.8	3.6	**10.1**	0.6	2.8	0.0	1.3	0.3	3.4	0.2	4.5
**QTL037**	AVR-Lc-03698.03-152405578	3	A	G	0.057	0.4	0.6	0.0	1.6	0.3	0.1	0.7	0.7	0.6	4.7	0.0

**QTL037**	AVR-Lc-03712.03-154837989	3	G	A	0.064	0.3	0.0	0.0	1.4	0.3	0.1	1.4	0.8	0.5	5.0	0.1
**QTL038**	AVR-Lc-03869.03-192934047	3	G	A	0.489	0.7	1.5	5.0	0.1	0.9	1.5	0.9	1.5	0.0	0.4	0.2
**QTL039**	AVR-Lc-03983.03-230295656	3	C	T	0.074	0.1	**7.5**	0.4	1.3	0.0	1.0	0.2	0.6	0.0	0.1	0.2
**QTL039**	AVR-Lc-03987.03-231578053	3	C	A	0.074	0.1	**9.3**	0.6	1.3	0.2	1.1	1.0	0.5	2.2	0.1	1.9
**QTL033**	AVR-Lc-04030.03-023938415	3	G	A	0.058	0.4	0.7	0.1	0.3	0.6	0.8	0.4	2.2	0.1	4.1	0.1
**QTL040**	AVR-Lc-04137.03-269539529	3	C	A	0.071	0.2	2.5	0.5	1.4	0.3	0.3	0.7	0.4	1.2	0.1	4.1
**QTL034**	AVR-Lc-04656.03-038315503	3	A	G	0.12	0.2	**5.6**	2.7	1.4	1.5	0.6	0.7	0.3	0.3	0.7	0.5
**QTL041**	AVR-Lc-04751.03-400242285	3	G	A	0.225	1.5	1.5	0.6	0.4	2.6	2.4	4.0	0.9	4.3	0.6	3.4
**QTL042**	AVR-Lc-04888.03-426261315	3	T	C	0.093	0.1	0.4	0.5	0.0	0.2	4.6	0.1	0.1	0.2	0.3	0.4
**QTL035**	AVR-Lc-05053.03-072044999	3	G	A	0.382	0.1	0.8	0.1	4.2	0.1	0.2	0.4	1.6	0.3	1.5	0.2
**QTL036**	AVR-Lc-05067.03-074793162	3	A	G	0.191	0.1	0.0	0.1	1.6	0.1	0.8	1.5	4.7	0.2	0.1	0.1
**QTL036**	AVR-Lc-05096.03-082600182	3	C	T	0.191	0.1	0.0	0.0	1.5	0.0	0.6	1.4	4.1	0.0	0.1	0.1
**QTL047**	AVR-Lc-05145.04-104990751	4	G	A	0.091	0.8	0.6	0.9	1.0	0.2	0.1	0.8	0.1	0.5	4.8	0.2
**QTL047**	AVR-Lc-05152.04-107024094	4	G	A	0.081	0.1	0.0	0.3	0.3	0.7	0.0	0.2	0.0	0.1	5.1	0.2
**QTL047**	AVR-Lc-05161.04-109440416	4	G	A	0.092	0.2	0.2	0.5	0.4	0.4	0.1	0.3	0.1	0.2	4.4	0.1
**QTL047**	AVR-Lc-05167.04-112177418	4	T	C	0.109	0.8	0.9	2.5	0.3	1.2	0.5	1.1	0.1	1.1	4.4	0.3
**QTL047**	AVR-Lc-05182.04-116949401	4	C	T	0.094	0.7	0.4	0.9	0.7	0.3	0.1	0.7	0.1	0.5	4.4	0.2
**QTL047**	AVR-Lc-05192.04-119227034	4	T	C	0.084	1.0	0.6	0.9	0.8	0.4	0.1	0.6	0.1	0.6	5.0	0.3
**QTL047**	AVR-Lc-05203.04-122734802	4	C	T	0.063	0.4	1.3	2.7	0.3	0.5	0.1	1.5	0.1	0.5	**8.1**	0.8
**QTL047**	AVR-Lc-05259.04-144601100	4	T	C	0.063	0.2	0.0	0.4	1.1	0.4	0.2	0.2	0.2	0.1	4.6	0.5
**QTL047**	AVR-Lc-05266.04-145430883	4	A	G	0.103	1.0	0.6	0.7	0.7	0.5	0.3	1.1	0.0	1.4	4.9	1.4
**QTL047**	AVR-Lc-05278.04-148771802	4	C	T	0.102	0.2	0.0	0.1	0.6	1.0	0.1	0.3	0.1	0.7	4.0	1.7

**QTL047**	AVR-Lc-05287.04-150464062	4	T	C	0.087	0.4	0.4	0.6	0.8	0.6	0.3	0.2	0.0	0.2	4.6	0.1
**QTL047**	AVR-Lc-05296.04-152490575	4	T	C	0.089	0.2	0.1	0.1	0.7	0.2	0.2	0.2	0.1	0.1	4.9	0.2
**QTL047**	AVR-Lc-05305.04-155525631	4	T	C	0.087	0.4	0.2	0.5	0.6	0.6	0.2	0.2	0.0	0.2	4.6	0.1
**QTL047**	AVR-Lc-05322.04-163251061	4	G	A	0.08	0.4	0.1	0.4	0.9	0.3	0.0	1.0	0.2	0.4	**5.4**	0.3
**QTL047**	AVR-Lc-05328.04-165504278	4	C	T	0.088	0.3	0.3	0.6	0.4	0.3	0.2	0.2	0.1	0.2	4.8	0.2
**QTL043**	AVR-Lc-05340.04-001679490	4	T	C	0.078	1.7	0.3	0.0	0.2	0.1	1.0	0.5	0.1	1.1	4.4	0.8
**QTL044**	AVR-Lc-05454.04-021398009	4	C	T	0.056	0.3	**5.6**	0.4	3.2	0.7	0.4	0.4	0.2	0.5	0.0	0.3
**QTL048**	AVR-Lc-05529.04-243778031	4	C	T	0.438	0.5	4.5	2.4	0.3	2.5	0.2	0.5	0.7	0.2	0.5	0.0
**QTL049**	AVR-Lc-05740.04-302184757	4	G	A	0.073	0.3	4.5	**5.4**	1.1	1.4	0.3	0.4	0.6	0.4	0.1	0.1
**QTL050**	AVR-Lc-05789.04-315309984	4	G	A	0.059	0.6	0.5	0.4	0.4	0.0	0.1	1.6	1.3	4.7	0.2	3.5
**QTL051**	AVR-Lc-05801.04-318079189	4	G	A	0.072	0.1	**6.7**	0.1	2.9	1.1	0.9	1.0	0.7	0.8	0.0	1.1
**QTL052**	AVR-Lc-06098.04-394816044	4	G	T	0.054	1.0	0.3	0.3	0.9	0.1	0.8	4.5	0.9	2.4	0.2	0.7
**QTL053**	AVR-Lc-06116.04-399656942	4	G	T	0.183	0.2	0.2	0.2	1.2	4.6	0.0	0.5	0.5	0.4	0.2	0.0
**QTL054**	AVR-Lc-06170.04-416219290	4	C	A	0.116	0.3	4.1	0.1	0.8	1.7	0.8	0.1	0.1	0.4	0.1	0.6
**QTL055**	AVR-Lc-06265.04-438198992	4	C	T	0.417	1.5	0.7	0.4	4.1	0.8	1.6	0.3	0.6	0.1	0.3	0.2
**QTL056**	AVR-Lc-06326.04-449185572	4	C	A	0.111	1.0	0.0	0.2	0.4	0.2	0.5	1.0	1.0	0.3	4.3	0.7
**QTL057**	AVR-Lc-06339.04-451674618	4	C	A	0.071	0.2	0.3	0.9	0.1	0.2	0.4	0.9	0.9	0.2	**5.8**	0.6
**QTL057**	AVR-Lc-06341.04-451714023	4	G	T	0.062	0.2	0.3	0.2	0.2	0.2	0.3	1.4	0.3	0.1	**5.7**	0.5
**QTL058**	AVR-Lc-06433.04-469775636	4	G	A	0.355	0.2	1.0	0.5	4.3	3.6	0.1	0.4	0.6	0.5	0.3	1.2
**QTL059**	AVR-Lc-06444.04-471058793	4	G	A	0.127	0.5	4.7	1.2	2.4	0.1	0.3	0.5	0.1	0.1	0.0	0.1
**QTL045**	AVR-Lc-06527.04-050552783	4	A	C	0.094	0.9	0.0	0.2	0.1	0.1	0.3	0.4	0.8	0.7	**7.6**	0.3
**QTL047**	AVR-Lc-06633.04-084622211	4	G	A	0.078	0.1	0.0	0.0	0.1	0.6	0.1	0.4	0.0	0.2	4.8	0.2

**QTL047**	AVR-Lc-06644.04-089591157	4	G	A	0.092	1.0	0.4	0.9	0.7	0.1	0.1	1.3	0.1	0.8	4.9	0.4
**QTL047**	AVR-Lc-06652.04-092451825	4	T	C	0.08	1.6	0.6	1.0	0.8	0.5	0.1	1.4	0.2	1.5	**5.2**	0.6
**QTL046**	AVR-Lc-06657.04-094813562	4	G	A	0.303	1.3	0.4	0.8	0.0	0.1	0.6	1.2	0.1	4.1	0.4	0.8
**QTL047**	AVR-Lc-06659.04-095095176	4	A	G	0.084	0.4	0.2	0.8	0.2	0.6	0.4	0.4	0.0	0.6	4.8	0.1
**QTL046**	AVR-Lc-06666.04-097561437	4	T	C	0.448	1.7	0.2	0.1	0.1	0.4	1.1	1.5	0.1	4.1	1.2	1.3
**QTL047**	AVR-Lc-06671.04-099280552	4	A	G	0.094	0.4	0.1	0.1	0.9	0.1	0.3	0.3	0.1	0.2	4.2	0.5
**QTL061**	AVR-Lc-06725.05-011593595	5	C	T	0.09	0.2	**6.0**	4.6	0.4	0.1	0.2	0.2	0.7	1.9	0.1	0.5
**QTL065**	AVR-Lc-06798.05-144806665	5	C	T	0.389	2.8	0.3	0.3	0.5	0.9	0.4	1.4	0.3	4.3	0.3	3.1
**QTL062**	AVR-Lc-06969.05-022095933	5	A	C	0.071	0.4	0.1	1.9	0.7	2.2	0.4	**5.2**	0.9	2.7	0.2	1.0
**QTL063**	AVR-Lc-06971.05-022411138	5	A	C	0.091	0.0	4.9	0.1	0.3	0.5	0.4	0.1	0.5	0.0	0.1	0.1
**QTL064**	AVR-Lc-07086.05-026835995	5	T	G	0.06	0.0	0.1	0.2	0.2	0.0	0.7	0.7	0.7	0.3	**6.7**	0.1
**QTL066**	AVR-Lc-07199.05-317998176	5	C	A	0.117	0.2	1.6	0.5	0.2	0.1	0.6	0.1	0.0	0.6	0.1	4.7
**QTL067**	AVR-Lc-07474.05-420462935	5	C	T	0.071	0.4	0.1	0.1	0.2	0.7	0.6	0.5	0.6	0.3	**5.6**	0.6
**QTL067**	AVR-Lc-07483.05-422569016	5	G	A	0.075	0.1	0.1	0.1	0.6	0.8	0.6	0.3	0.2	0.0	5.0	0.5
**QTL067**	AVR-Lc-07494.05-425439183	5	G	T	0.128	0.6	0.1	0.5	0.4	0.3	0.4	1.5	0.3	1.3	4.3	0.7
**QTL068**	AVR-Lc-07604.05-441955698	5	G	A	0.091	0.8	0.5	4.2	2.1	0.6	0.3	0.7	0.5	0.7	0.1	1.1
**QTL069**	AVR-Lc-07771.05-470073827	5	C	T	0.054	0.0	0.9	2.7	0.1	4.2	0.4	0.6	0.1	1.4	0.3	0.9
**QTL060**	AVR-Lc-07900.05-008235645	5	A	C	0.089	0.9	0.2	0.7	0.7	0.7	0.0	0.4	0.1	0.0	**5.9**	0.2
**QTL070**	AVR-Lc-08000.06-011687720	6	T	G	0.456	0.2	4.1	2.8	0.2	0.3	0.1	0.1	0.5	0.2	0.4	0.8
**QTL071**	AVR-Lc-08010.06-011899372	6	C	T	0.053	0.1	3.1	**8.0**	0.9	0.8	0.8	0.7	0.1	1.2	0.0	2.0
**QTL074**	AVR-Lc-08166.06-172835867	6	C	A	0.056	0.0	0.6	1.6	0.9	0.7	0.4	0.5	0.1	1.4	0.1	4.3
**QTL075**	AVR-Lc-08209.06-184525439	6	T	C	0.061	0.1	4.9	0.4	0.3	2.1	0.5	0.2	0.2	0.2	0.1	0.1

**QTL075**	AVR-Lc-08213.06-186928146	6	C	T	0.075	0.1	4.4	0.4	0.3	2.1	0.4	0.3	0.5	0.1	0.1	0.1
**QTL076**	AVR-Lc-08292.06-221642340	6	C	T	0.07	0.3	0.3	0.4	1.0	0.1	0.4	0.2	0.0	0.4	**5.4**	1.3
**QTL076**	AVR-Lc-08300.06-223824687	6	G	A	0.078	0.2	0.2	0.3	1.1	0.0	0.4	0.1	0.0	0.3	5.0	1.1
**QTL072**	AVR-Lc-08587.06-031922562	6	G	A	0.094	0.7	4.6	0.1	0.1	0.5	0.6	0.6	0.1	2.8	0.3	1.1
**QTL078**	AVR-Lc-08637.06-328113285	6	C	T	0.093	0.6	1.4	0.1	0.9	1.2	0.1	0.7	0.2	2.6	0.2	5.1
**QTL077**	AVR-Lc-08717.06-348699147	6	G	A	0.077	0.3	0.1	0.2	0.1	0.1	0.2	0.1	0.3	0.9	**5.4**	0.1
**QTL078**	AVR-Lc-08863.06-381679296	6	T	G	0.162	0.3	0.9	1.0	0.3	0.0	0.0	0.6	0.4	0.9	0.0	4.5
**QTL078**	AVR-Lc-08869.06-382855264	6	A	G	0.153	0.3	1.1	1.3	1.2	0.0	0.0	0.2	0.5	1.0	0.1	4.7
**QTL079**	AVR-Lc-08870.06-382869448	6	G	A	0.345	0.2	1.0	1.5	5.0	0.5	0.3	0.4	0.1	0.2	0.3	1.9
**QTL080**	AVR-Lc-09049.06-413154045	6	G	A	0.226	4.2	0.7	0.1	0.1	0.2	0.4	1.0	0.6	0.5	0.4	0.3
**QTL080**	AVR-Lc-09063.06-414723087	6	A	G	0.166	4.5	0.8	0.3	0.6	0.3	0.6	0.5	1.5	1.4	0.5	0.6
**QTL081**	AVR-Lc-09086.06-418350448	6	G	A	0.053	1.0	0.3	1.8	4.3	0.1	0.2	0.4	0.9	0.6	0.2	0.5
**QTL082**	AVR-Lc-09103.06-419840563	6	A	G	0.188	0.8	0.3	0.2	4.3	0.2	0.2	0.5	1.2	0.1	0.2	0.3
**QTL073**	AVR-Lc-09234.06-084823796	6	A	G	0.419	0.7	1.4	0.1	0.8	0.4	0.2	0.9	0.3	1.8	0.2	4.3
**QTL083**	AVR-Lc-10007.07-447269681	7	C	T	0.368	0.6	0.2	0.7	0.4	**5.4**	0.4	0.6	0.5	0.3	0.3	0.4
**QTL084**	AVR-Lc-10129.07-487701700	7	A	C	0.334	0.6	0.0	0.1	4.4	0.4	0.4	0.4	2.0	0.3	0.3	1.0
**QTL085**	AVR-Lc-10391.07-528906358	7	G	A	0.469	0.5	0.3	0.1	0.6	1.7	0.1	4.1	0.6	0.4	1.1	0.4

QTL: quantitative trait loci, SNP: single nucleotide polymorphism, Chr: Chromosome, MAF: minor allele frequency, HDS2: second herbicide damage score, DFLR: days to flowering, RI_{DFLR}: DFLR reduction index, DMAT: Days to maturity, RI_{DMAT}: DMAT reduction index, PH: Plant height, BY: Biological yield per plant, SY: seed yield per plant, NPP: number of pods per plant, RI_{NPP}: NPP reduction index, NSP: number of seeds per plant.
